# The combined effect of social pensions and cash transfers on child mortality: evaluating the last two decades in Brazil and projecting their mitigating effect during the global economic crisis

**DOI:** 10.1016/j.lana.2023.100618

**Published:** 2023-11-03

**Authors:** Temidayo James Aransiola, José Alejandro Ordoñez, Daniella Medeiros Cavalcanti, Gabriel Alves de Sampaio Morais, Dandara de Oliveira Ramos, Davide Rasella

**Affiliations:** a Institute of Collective Health (ISC) at the Federal University of Bahia (UFBA), Bahia, Brazil; b Institute of Global Health (ISGlobal), Barcelona, Spain

**Keywords:** Social pension, Cash transfer programs, Social protection, Child mortality, Global economic crisis, Multiple crisis, Fiscal austerity, Poverty, Impact evaluation, Microsimulation models, Forecasting

## Abstract

**Background:**

The world is currently experiencing multiple economic crises due to the COVID-19 pandemic, war in Ukraine, and inflation surge, which disproportionately affect children, especially in low- and middle-income countries (LMICs). We evaluated if the expansion of Social Assistance, represented by Social Pensions (SP) and Conditional Cash Transfers (CCT), could reduce infant and child mortality, and mitigate excess deaths among children in Brazil, one of the LMICs most affected by these economic crises.

**Methods:**

We conducted a retrospective impact evaluation in a cohort of Brazilian municipalities from 2004 to 2019 using multivariable fixed-effects negative binomial models, adjusted for relevant demographic, social, and economic factors, to estimate the effects of the SP and CCT on infant and child mortality. To verify the robustness of the results, we conducted several sensitivity and triangulation analyses, including difference-in-difference with propensity-score matching. These results were incorporated into dynamic microsimulation models to generate projections to 2030 of various economic crises and Social Assistance scenarios.

**Findings:**

Consolidated coverage of SP was associated with significant reductions in infant and child mortality rates, with a rate ratio (RR) of 0.843 (95% CI: 0.826–0.861) and 0.840 (95% CI: 0.824–0.856), respectively. Similarly, CCT consolidated coverages showed RRs of 0.868 (95% CI: 0.842–0.849) and 0.874 (95% CI: 0.850–0.899) for infant and child mortality, respectively. The higher the degree of poverty in the municipalities, the stronger the impact of CCT on reducing child mortality. Given the current economic crisis, a mitigation strategy that will increase the coverage of SP and CCT could avert 148,736 (95% CI: 127,148–170,706) child deaths up to 2030, compared with fiscal austerity measures.

**Interpretation:**

SP and CCT programs could strongly reduce child mortality in LMICs, and their expansion should be considered as an effective strategy to mitigate the impact of the current multiple global economic crises.

**Funding:**

10.13039/100000865Bill & Melinda Gates Foundation, Grant_Number:INV-027961. 10.13039/501100000265Medical Research Council(MRC-UKRI),Grant_Number:MC_PC_MR/T023678/1.

**Research in context**

**Evidence before this study**

To investigate the available evidence on the impact of cash transfers and social pensions on child mortality, we conducted a comprehensive search of the PubMed database for studies published in English that contained the following terms: “cash transfers” [MeSH Terms] OR “social pensions” [MeSH Terms] AND [“infant mortality” [MeSH Terms] OR “child mortality” [MeSH Terms]]. Our search was not restricted by language or date, and the most recent search was conducted in September 2022. Additionally, we reviewed the reference lists of selected articles to identify any relevant studies that may have been missed.

No studies have ever evaluated the effects of Social Pensions on child survival or mortality. On the other side, our search yielded a number of studies that examined the effects of cash transfers on child and infant mortality, with many reporting a positive impact on mortality reduction in low- and middle-income countries (LMIC), particularly with respect to poverty-related diseases such as diarrhoea, malnutrition, and lower respiratory tract infections. However, many of these studies only examined data up to 2010 or 2015, and there is a relative paucity of updated evidence on this topic.

Furthermore, to our knowledge, no research has been published on the combined impact of social pensions and cash transfers and on all-cause, all-age child mortality on a national scale. Additionally, no study has thus far utilized cohort data to capture the effects of implementing these programs, or forecasted the mitigation effects of both programs in periods of economic crises.

**Added value of this study**

The robust evaluation, with a large cohort for a period of almost two decades, of the combined impact of Social Pensions and Conditional Cash Transfers, which represent the backbone of Social Assistance in LMIC, on infant and child mortality, using a rigorous approach that combine advanced mathematical and econometric techniques. Moreover, the integration of the retrospective analyses, with their datasets and effects estimated, with dynamic microsimulation models to generate projections until 2030, taking into account various combinations of economic crises and alternative policy responses.

**Implications of all the available evidence**

Our study provides compelling evidence that the implementation of Social Pensions programs, in combination with sizeable Conditional Cash Transfer programs, can effectively reduce infant and child mortality in LMIC. Moreover, their expansion should be considered an effective strategy to mitigate the impact of the current global economic crisis, while their reduction due to fiscal austerity measures would be extremely prejudicial to child health.

## Introduction

The combined impact of the COVID-19 pandemic, the war in Ukraine and the inflationary crisis has resulted in the largest global increase in poverty, inequality, and food insecurity in decades, with children disproportionately affected.^1^ It is estimated that these multiple global crises have lead to an additional 75–95 million people living in extreme poverty, reaching a total of almost 700 million people, of which 356 million are children.^2^^,^^3^ At the present time, over a billion children are living in multidimensional poverty—deprived of key dimensions of childhood such as health, education and nutrition, while 1.5 billion children below 15 years have no access to social protection.^3^ Children are suffering one of the worst hunger crisis in decades, with the need of urgent mitigation interventions.^4^ The current context of multiple crises has pushed the world further off track from the global goal of ending extreme poverty by 2030: given current trends, 574 million people—nearly 7 percent of the world's population—will still be living on less than $2.15 a day in 2030.^2^^,^^3^ Future economic scenarios are even more worrisome: it has been forecasted a deceleration of the economy and one of the lowest global growth rates in recent decades, with a looming global economic recession.^5^ As a consequence, poverty-related morbidity and mortality, in particularly in children, could substantially increase in LMICs if effective mitigation strategies are not promptly implemented.

The United Nations General Assembly's Sustainable Development Goals (SDGs) include Target 3.2, which calls for the eradication of avoidable deaths of newborns and children under the age of 5. The target aims for all nations to reduce neonatal mortality (NMR) to no higher than 12 per 1000 live births and under-5 mortality (U5MR) to no higher than 25 per 1000 live births by 2030.^6^ Although most countries have achieved SDG sub-target 3.2, global trends–estimated before the beginning of the recent multiple crises–indicate that 53 out of 195 countries risked missing the SDG U5MR, putting 48 million children under-5 at risk between 2020 and 2030.^7^

During periods of socioeconomic and health crises, Social Assistance, defined as non-contributory transfers to those individuals deemed eligible for assistance on the basis of their vulnerability or poverty, should provide a safety net for poor and excluded people.^8^ In the majority of LMIC, Social Assistance is provided by two main public policies: Conditional Cash Transfer (CCT) Programs and Social Pensions (SP).^8^ Recent reports from international organizations such as UNICEF, ILO, and Word Bank emphasize the importance of expanding existing Social Assistance programs to mitigate the harmful effects of the recent multiple health and economic crises, in particular on infant and child health, and warn about the application of impactful fiscal austerity measures in LMICs,^6^^,^^7^ which could have extremely harmful effects on the health of the most vulnerable.^9^

Brazil has developed during the last decades a structured strategy for Social Assistance, based of one of the world's largest CCT programs targeted at the poor population (*Programa Bolsa Família*-BFP),^10^ and one of the world's most comprehensive Social Pensions programs for the elderly and disabled population, the *Benefício de Prestação Continuada* (BPC).^11^ Moreover, Brazil is one of the LMICs where the COVID-19 pandemic had the strongest and most prolonged impact on the economy, with a rapid and sustained increase in poverty rates,^12^ that–during the post-pandemic period—have hit their highest peak in more than a decade, and food insecurity that reached 60% of the population, leaving more than 15% in hunger.^13^ While, in previous years, fiscal austerity measures have prevented the expansion of BFP and BPC, even during strong economic crises,^14^ there is currently the opportunity in the country to expand Social Assistance as safety net for the new poor created by the recent multiple crises.

No studies have ever evaluated the impact of SP on child health, and, while previous studies have shown the importance of CCTs in decreasing poverty-related child mortality,^15^, ^16^, ^17^ none has ever estimated the joint effects of these two Social Assistance policies in periods of economic crises.

The aim of this study was to evaluate the combined impact of Social Protection and Conditional Cash Transfer programs over the last two decades on infant and child mortality rates in Brazil, forecasting a range of potential economic crises scenarios up to 2030, and estimating the mitigation effects of alternative implementations of SP and CCT, ranging from expansions for covering the new poor to reductions due to fiscal austerity measures.

## Methods

### Study design

This study used a longitudinal ecological design to analyze the relationship between child mortality, BFP and BPC coverages in Brazilian municipalities from 2004 (year of the BFP implementation, while BPC was already existing) to 2019. To ensure data quality, 2548 municipalities with adequate vital statistics out of the total of 5507 were included in the analysis, following the established methodology of previous studies.^16^^,^^18^ The dependent variables were age-specific mortality rates for three groups: under-1 year, from 1 to 4 years, and under-5 years.

The BFP coverage was determined by dividing the number of families enrolled in the program by the eligible population, which comprised poor households according to the BFP poverty lines. The BFP coverage was categorized into established thresholds in the literature to estimate the dose–response effect associated with the expansion of the program, as in previous studies^16^^,^^18^: low (0–29.9%), intermediate (30–69.9%), high (70–99.9%), and consolidated (≥100%). For the BPC, which lacked established thresholds in the literature, coverage was categorized into terciles: low (0–32.9 percentile), intermediate (33–65.9 percentile), and consolidated (66–100 percentile). All the relevant demographic, socioeconomic, and healthcare adjusting variables according to the literature were included in the models^15^, ^16^, ^17^, ^18^: poverty rates, illiteracy rates, urbanization rates, fertility rates, percentage of households with inadequate garbage collection, hospital bed rates per 1000 population, number of physicians per 1000 population, and number of nurses per 1000 population. A wide range of other potential confounding covariates was also tested as sensitivity analyses (Appendix, p. 8–18). Due to the categorization of the exposure variables, and following the approach of previous studies,^15^^,^^16^^,^^18^ the adjusting variables were dichotomized using their median values over the period. Year binary variables were included to control for time-specific shocks, representing the major economic and political crises in Brazil (2008–09, 2013–14, and 2015–16). Other unobserved time-invariant characteristics of the municipalities were adjusted by the fixed effects term.^19^

### Data sources

The data used in this study were obtained from multiple sources. Health outcome and service data (deaths, hospital beds, physicians, and nurses) were collected from the Ministry of Health, while data on BFP and BPC benefits were obtained from the Ministry of Social Development. Adjusting socioeconomic and demographic variables were sourced from surveys and censuses conducted by the Brazilian Institute of Geography and Statistics. The complete list of data sources with references, alongside the detailed methods for data interpolation and extrapolation, are presented in the Appendix, Web-Table 1.

### Role of the funding source

The sponsor of the study had no role in study design, data collection, data analysis, data interpretation, or writing of the report.

### Statistical analyses

#### Retrospective analysis

The study used fixed-effect models estimated through negative binomial regression method, following previous ecological impact evaluation studies.^16^^,^^18^^,^^20^ The negative binomial distribution^21^^,^^22^ was chosen because municipal mortality rates are characterized by overdispersion,^16^^,^^18^^,^^20^ and the fixed-effect specification was selected through the Hausman test,^23^ and because it enables controlling for time-invariant unobserved determinants of mortality that could also be associated with interventions coverage, such as historical, geographical, or sociocultural characteristics of each municipality.^18^

We performed several sensitivity analyses to validate the robustness of the results. First, to verify the existence of omitted-variable bias, a wide range of time-variant potential confounding variables were added to the model while observing the changes in the estimates for the main exposures. Second, to assess the effects of categorizations and dichotomizations, the models were estimated using continuous variables. Third, to perform an external validation of our estimates obtained through sample selection, we fit the models using the entire 5507 Brazilian municipalities. Fourth, to assess the relevance of the starting period, the models were also estimated using data from 2000 to 19 (2004 was chosen as starting year because BFP was not present before, but similar CCT programs—without available data—were already implemented). Fifth, to assess the potential collinearity between poverty levels and the SP and CCT coverages, the models were estimated without poverty. Sixth, to verify the influence of the specific time shocks, several sets of alternative time controls were tested. Seventh, to evaluate the coherence of the overall results with an alternative empirical strategy, the models were estimated using Poisson regression and the results were compared using the Akaike Information Criterion (AIC) and Bayesian Information Criterion (BIC). The last robustness check was the estimation of models for external causes of death (V01–V98, from International Classification of Diseases, 10th revision—ICD-10), using the same specification, expecting no significant effect of BPC and BFP, i.e. acting as a negative control.^18^^,^^20^ Finally, to have a higher degree of confidence in the causal inference and in the impact evaluation, we did triangulation analyses evaluating the effects of BFP and BPC on U5MR by diff-in-diff with PSM,^24^ using the municipalities with no or low coverage of these programs since 2004 (n = 602), versus medium and high coverage since 2004 (1944), analyzed in the years 2004 and 2019. The findings reported in this study withstood all the sensitivity and triangulation analyses, showing that our results are robust and with an high degree of confidence (see pages 8–18 in the Appendix). The Stata version 14 (Stata Corp, College Station)^25^ was used for data processing and analysis.

#### Forecasting and future scenarios

We used validated municipal-level microsimulation models to forecast the impact on under-5 mortality rates of potential economic crisis scenarios combined with alternative implementations of BPC and BPF. Microsimulation is considered one of the most accurate forecasting methods because it allows to model municipality-specific characteristics and their associated outcome probabilities.^26^ The modelling was performed in two stages: first, we created a synthetic cohort of all Brazilian municipalities for 2020–2030 by extrapolating and modelling the independent variables from the 2004 to 19 dataset. Second, we predicted the mortality rates from 2020 to 30 using the independent variables, the estimates from the first stage and the regression models of the retrospective analyses.

In the first stage of our analysis, we simulated three economic crisis scenarios of short, medium, and long duration. These scenarios were based on changes in poverty rates calculated using microdata from national household surveys conducted during the COVID-19 pandemic, and were extrapolated using exponential decay functions from previous studies.^14^^,^^27^ To address these crisis scenarios, we simulated changes in the BFP and BPC coverages through three policy response scenarios: the mitigation scenario, the baseline scenario, and the severe fiscal austerity scenario. The mitigation scenario involves increasing BFP and BPC coverages proportionally to the increase of poverty rates during the economic crisis, followed by coverage reductions after the crisis peak.^14^^,^^27^ The baseline scenario involves maintaining the current Brazilian fiscal austerity rules (Emenda Constitucional 95), slowly reducing the BFP and BPC coverages even during the economic crisis (details in p. 19 of the Appendix),^28^ as already modelled in previous studies.^14^^,^^27^ The severe austerity scenario involves reducing the BFP and BPC coverages proportionally to the reduction observed in government expenditure on social protection (excluding cash transfer programs) from 2014 to 19.^29^ We performed 10,000 Monte Carlo simulations for each outcome and scenario, ensuring the variation of the parameter values in each simulation cycle. The modelling procedures were following the international model reporting guidelines (ISPOR-SMSM),^30^ and models calibration, internal and external validation, parameter distributions for Monte Carlo simulations, and models equations are provided in the Appendix p. 21. For the forecasting analysis, we used R Statistical software (version 4.1.2).^31^

## Results

### Retrospective analysis

**Fig. 1:**
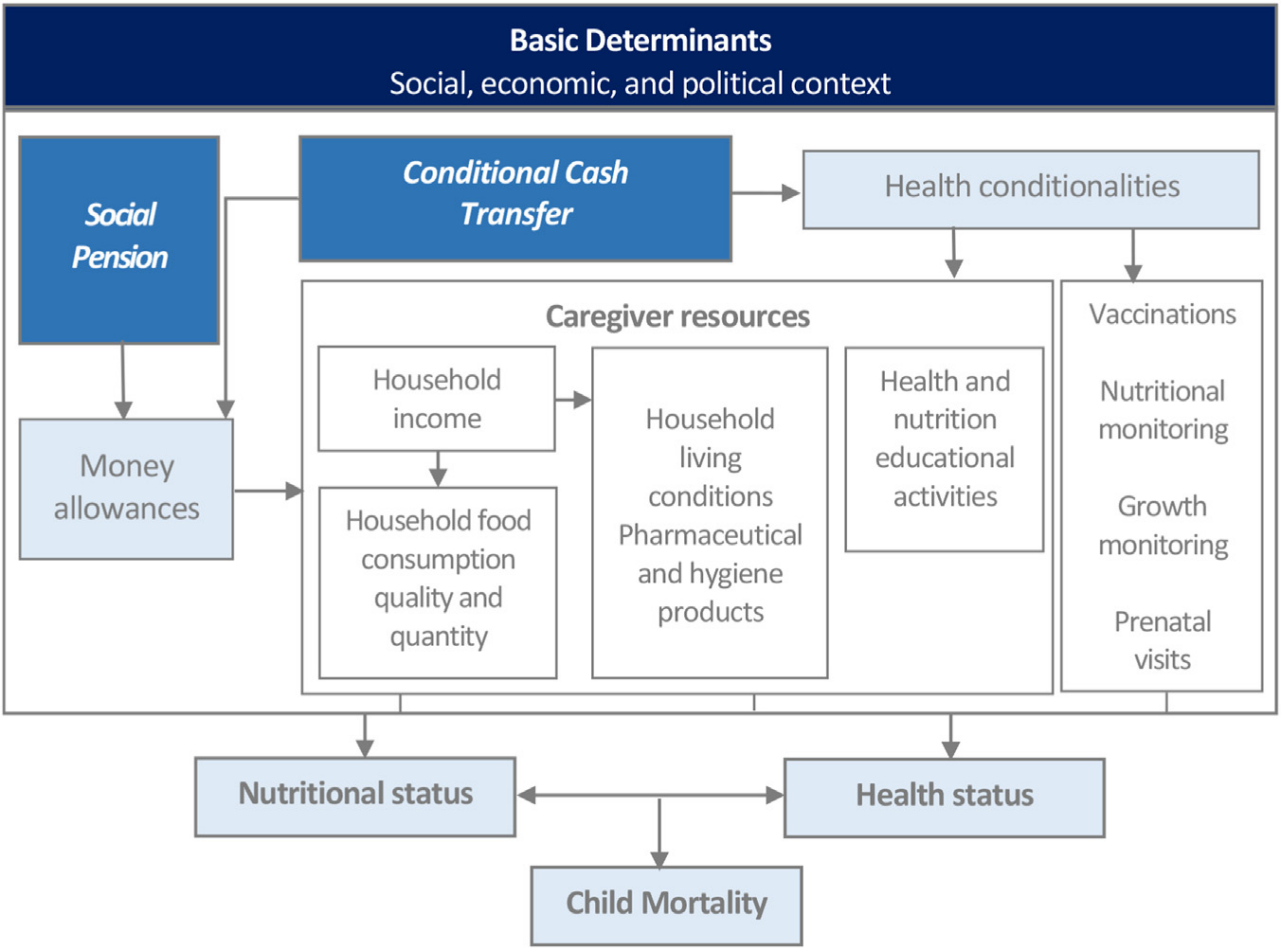
**Mechanisms linking the social pension program, bolsa familia program and the family health program to health outcomes**.

Table 1 shows the descriptive statistics for the variables included in this study, and for 2548 municipalities with adequate vital statistics. Between 2004 and 2019, there was a significant reduction in under-1 and under-5 mortality rates, decreased by 35% and 33%, respectively. During the same time period, the target coverage of the BFP amost doubled, increasing sharply from 2004 and then stabilizing, while the municipal coverage of the BPC, which was lower than that of the BFP, also steadily increased by 89%. Socioeconomic, healthcare, and living conditions improved overall during the study period. Fig. 1 shows the framework mechanisms that could explain the effects of each intervention on child health. Table 2 shows the crude and adjusted models for mortality rates for children under-1, from 1 to 4, and under-5, focusing on the association with the coverage levels of the BFP and BPC. In the adjusted models, consolidated coverages of BFP were significantly associated with reduced incidence rates for under-1, 1–4, and under-5 mortality, with Rate-Ratios (RR) of 0.868(95% CI: 0.842–0.894), 0.914(95% CI: 0.854–0.979), and 0.874 (95% CI: 0.850–0.899), respectively. Similarly, the coverage of the BPC was associated with significant reductions in the same mortality rates, with RR of 0.843(95% CI: 0.826–0.861), 0.822(95% CI: 0.783–0.862), and 0.840 (95% CI: 0.824–0.856), respectively. At all levels of coverage, the mitigating effect of the BFP on mortality was higher among children under-1, while the BPC had a greater impact on reducing mortality among children aged 1–4 years old. All sensitivity and triangulation analyses, including Diff-in-diff with PSM, confirmed the robustness of the results, and are presented, with additional complementary tests, in the Appendix on pages 8–18.

**Table 1:** Infant mortality rates, socioeconomic, demographic and health variables for selected municipalities (N = 2548), from 2004 to 2019.

Variables	2004		2009		2014		2019		%Δ
	Mean	s. d.	Mean	s. d.	Mean	s. d.	Mean	s. d.	2004–2019
Mortality rate for children younger than 5 years per 1000 livebirths (%)									
Infant (Under 1 years)	18.20	(12.95)	14.67	(12.53)	12.59	(10.94)	12.26	(10.85)	−32.66%
Toddler (1–4 years)	3.20	(5.27)	2.75	(5.77)	2.09	(4.21)	2.09	(4.79)	−34.75%
Child (Under 5 years)	21.40	(14.48)	17.41	(14.23)	14.68	(12.23)	14.34	(12.05)	−32.97%
BFP Coverage (%)	48.10	(23.23)	99.45	(3.67)	99.90	(1.42)	97.69	(9.58)	103.07%
BPC Coverage (%)	1.08	(0.77)	1.48	(1.00)	1.86	(1.15)	2.04	(1.22)	89.02%
Proportion of individuals older than 15 years who are illiterate (%)	15.39	(9.66)	13.02	(8.03)	11.33	(7.21)	8.78	(6.58)	−42.96%
Poverty rate (%)	25.53	(18.47)	16.39	(15.25)	9.93	(12.69)	10.15	(13.60)	−60.26%
Urbanization rate (%)	66.55	(20.76)	68.82	(20.08)	72.12	(19.71)	74.11	(19.63)	11.36%
Fertility rate (%)	3.63	(0.65)	3.36	(0.55)	3.07	(0.47)	2.89	(0.45)	−20.45%
Rate of hospital beds per 1000 population	2.60	(2.73)	2.34	(2.40)	2.16	(2.19)	2.09	(2.29)	−19.63%
Rate of physicians per 1000 population	0.68	(0.51)	0.69	(0.60)	0.79	(0.69)	0.90	(0.81)	32.02%
Rate of nurses per 1000 population	0.36	(0.22)	0.49	(0.23)	0.73	(0.35)	0.94	(0.53)	161.84%
Households with proper garbage collection	66.88	(21.95)	75.90	(19.08)	82.51	(17.57)	86.73	(16.22)	29.68%

**Note**: Data are mean (SD). The mortality rates are calculated by 1000 livebirths. BFP = Bolsa Familia Programme. PBC = Benefício de Prestação Continuada Programme. SD = Standard Deviation.

**Table 2:** Incidence rate ratios from the fixed effect negative binomial models for the association between child and infant mortality rates and Bolsa Família Program (BFP) and Benefício de Prestação Continuada (BPC) coverage.

	Infant (Under 1)		Toddler (From 1 to 4)		Child (Under 5)	
	Crude	Adjusted	Crude	Adjusted	Crude	Adjusted
BFP target coverage						
Low (0–30%)	1	1	1	1	1	1
Intermediate (30–70%)	1.007 [0.975–1.039]	1.008 [0.976–1.041]	1.064 [0.990–1.144]	1.068 [0.993–1.148]	1.015 [0.985–1.046]	1.017 [0.987–1.047]
High (70–100%)	0.937∗∗∗ [0.908–0.967]	0.952∗∗∗ [0.922–0.983]	0.959 [0.892–1.030]	0.980 [0.912–1.053]	0.941∗∗∗ [0.913–0.969]	0.956∗∗∗ [0.928–0.985]
Consolidated (≥100%)	0.828∗∗∗ [0.803–0.853]	0.868∗∗∗ [0.842–0.894]	0.854∗∗∗ [0.799–0.914]	0.914∗∗∗ [0.854–0.979]	0.832∗∗∗ [0.809–0.856]	0.874∗∗∗ [0.850–0.899]
BPC municipal coverage						
Low (0–33%)	1	1	1	1	1	1
Intermediate (33–66%)	0.908∗∗∗ [0.896–0.920]	0.920∗∗∗ [0.908–0.933]	0.895∗∗∗ [0.867–0.924]	0.913∗∗∗ [0.884–0.943]	0.906∗∗∗ [0.894–0.917]	0.919∗∗∗ [0.907–0.931]
Consolidated (66–100%)	0.792∗∗∗ [0.777–0.808]	0.843∗∗∗ [0.826–0.861]	0.755∗∗∗ [0.721–0.790]	0.822∗∗∗ [0.783–0.862]	0.786∗∗∗ [0.772–0.801]	0.840∗∗∗ [0.824–0.856]
Proportion of individuals older than 15 years who are illiterate (%)		1.053∗∗∗ [1.032–1.075]		1.062∗∗ [1.013–1.114]		1.054∗∗∗ [1.034–1.075]
Poverty rate (%)		1.034∗∗∗ [1.018–1.050]		1.067∗∗∗ [1.029–1.107]		1.038∗∗∗ [1.023–1.053]
Urbanization rate (%)		0.934∗∗∗ [0.907–0.962]		0.925∗∗ [0.865–0.990]		0.933∗∗∗ [0.908–0.959]
Fertility rate (%)		1.057∗∗∗ [1.038–1.076]		1.039 [0.996–1.084]		1.054∗∗∗ [1.036–1.072]
Rate of hospital beds per 1000 population		1.002 [0.983–1.020]		1.001 [0.959–1.044]		1.001 [0.985–1.019]
Rate of physicians per 1000 population		0.984 [0.966–1.001]		1.009 [0.969–1.050]		0.987 [0.971–1.004]
Rate of nurses per 1000 population		0.933∗∗∗ [0.921–0.945]		0.898∗∗∗ [0.872–0.925]		0.928∗∗∗ [0.917–0.939]
Households with proper garbage collection		0.966∗∗∗ [0.945–0.987]		0.929∗∗∗ [0.885–0.976]		0.960∗∗∗ [0.941–0.979]
Year binaries	Yes	Yes	Yes	Yes	Yes	Yes
**Number of observations**	40,730	40,730	38,634	38,634	40,762	40,762
**AIC**	134,979.9	134,562.4	64,055.5	63,921.6	142,235.4	141,707.0
**BIC**	135,091.9	134,743.3	64,166.8	64,101.4	142,347.4	141,887.9
**Log-Likelihood**	−67,476.9	−67,260.2	−32,014.8	−31,939.8	−71,104.7	−70,832.5

**Note:** Data are in Rate Ratio (RR) coefficients (95% CI) unless otherwise specified. The confidence intervals are in parentheses. Time shocks are controls for specific years of economic crisis (2008, 2013 and 2015). The symbols ‘∗∗∗’ and ‘∗∗’ denote significance at 1% and 5%, respectively. BFP = Bolsa Familia Programme. PBC = Benefício de Prestação Continuada Programme.

Table 3 displays the findings of the under-5 mortality model, which is further analyzed by quartiles of poverty rates to examine how the effects of the BFP and BPC varies based on the poverty levels in each municipality. The results show that the BFP program had no effect in municipalities with the lowest poverty rates, but had the highest impact in those with the highest poverty rates, where the BFP consolidated coverage level is associated with a RR of 0.818 (95% CI: 0.776–0.863) for U5MR. Conversely, there is no clear trend observed in the effects of BPC coverage on child mortality across different poverty levels in the municipalities.

**Table 3:** Incidence rate-ratios from the fixed effect negative binomial models for the association between under-5 mortality rate and Bolsa Família Program (BFP) and Benefício de Prestação Continuada (BPC) coverage by poverty quartiles.

	Poverty quartile			
	Quartile 1	Quartile 2	Quartile 3	Quartile 4
BFP target coverage				
Low (0–30%)	1	1	1	1
Intermediate (30–70%)	3.479 [0.809–14.960]	0.969 [0.914–1.028]	1.001 [0.948–1.058]	1.013 [0.960–1.068]
High (70–100%)	2.847 [0.690–11.748]	0.965 [0.915–1.018]	0.962 [0.910–1.017]	0.927∗∗∗ [0.878–0.977]
Consolidated (≥100)	2.851 [0.696–11.674]	0.928∗∗∗ [0.882–0.975]	0.891∗∗∗ [0.845–0.940]	0.818∗∗∗ [0.776–0.863]
BPC municipal coverage				
Low (0–33%)	1	1	1	1
Intermediate (33–66%)	0.905∗∗∗ [0.869–0.944]	0.881∗∗∗ [0.865–0.898]	0.994 [0.956–1.034]	0.927∗∗∗ [0.897–0.958]
Consolidated (66–100%)	0.844∗∗∗ [0.792–0.900]	0.799∗∗∗ [0.772–0.826]	0.906∗∗∗ [0.863–0.951]	0.826∗∗∗ [0.790–0.863]
Proportion of individuals older than 15 years who are illiterate (%)	1.032 [0.946–1.126]	1.064∗∗ [1.012–1.119]	1.092∗∗∗ [1.060–1.125]	1.079∗∗ [1.005–1.158]
Urbanization rate (%)	0.971 [0.842–1.119]	0.905∗∗ [0.827–0.989]	0.971 [0.917–1.028]	0.933∗∗∗ [0.888–0.981]
Fertility rate (%)	1.115∗∗ [1.008–1.232]	1.053∗∗ [1.006–1.103]	1.063∗∗∗ [1.035–1.092]	1.117∗∗∗ [1.054–1.183]
Rate of hospital beds per 1000 population	0.998 [0.952–1.047]	0.994 [0.962–1.027]	1.046∗∗ [1.000–1.094]	1.005 [0.964–1.047]
Rate of physicians per 1000 population	0.970 [0.923–1.019]	1.031 [0.989–1.074]	1.005 [0.971–1.040]	0.974 [0.947–1.002]
Rate of nurses per 1000 population	0.959∗∗ [0.929–0.990]	0.939∗∗∗ [0.916–0.963]	0.941∗∗∗ [0.918–0.965]	0.929∗∗∗ [0.906–0.953]
Households with proper garbage collection	0.973 [0.885–1.069]	0.964 [0.903–1.029]	0.970 [0.931–1.010]	0.954∗∗ [0.914–0.995]
Year binaries	Yes	Yes	Yes	Yes
**Number of observations**	10,046	9991	9917	10,052
**AIC**	28,299.3	29,905.5	29,938.1	37,497.1
**BIC**	28,414.7	30,020.8	30,053.3	37,612.5
**Log-Likelihood**	−14,133.6	−14,936.7	−14,953.0	−18,732.5

**Note:** Data are in Rate Ratio (RR) coefficients (95% CI) unless otherwise specified. The confidence intervals are in parentheses. Time shocks are controls for specific years of economic crisis (2008, 2013, 2015 and 2018). The symbols ‘∗∗∗’ and ‘∗∗’ denote significance at 1% and 5%, respectively. BFP = Bolsa Familia Program. PBC = Benefício de Prestação Continuada Program.

### Forecasting

**Fig. 2:**
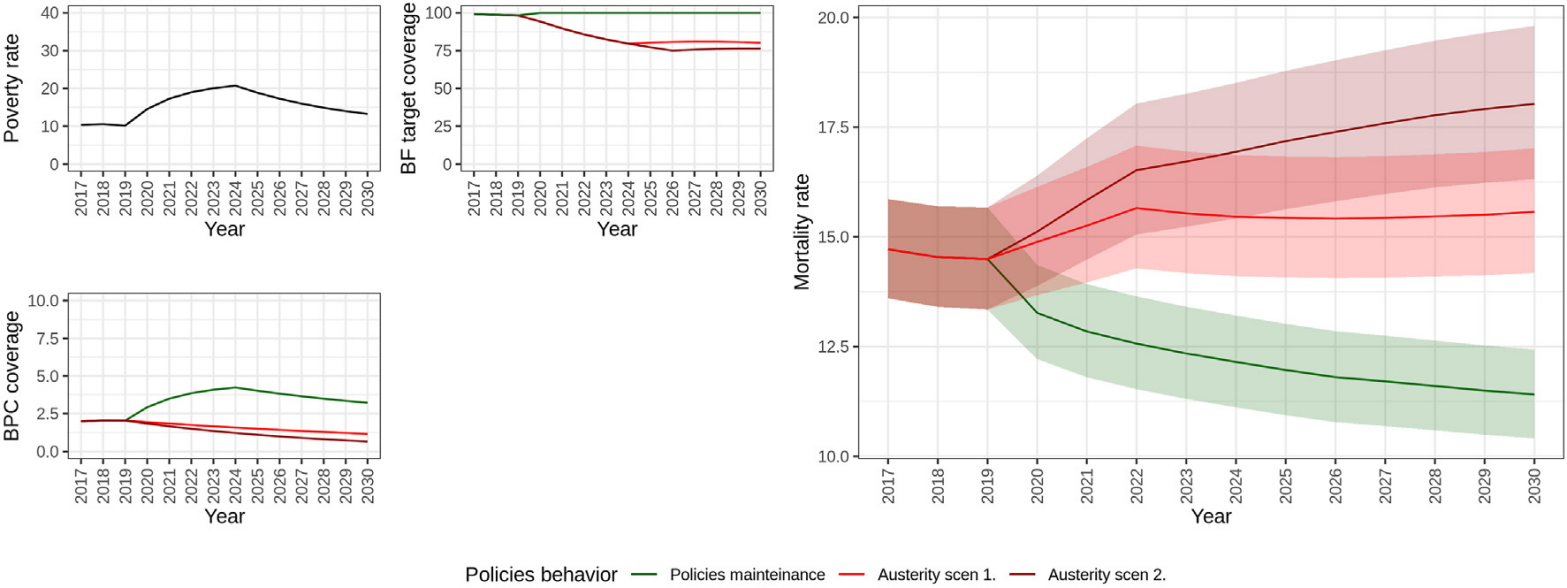
**Scenarios of poverty, cash transfer coverages, and under-5 mortality predictions, 2020–2030**. **Note**: The mortality rates are per 1000 livebirths. BF = Bolsa Familia Programme, PBC = Benefício de Prestação Continuada Programme.

Fig. 2 illustrates the results for the medium economic crisis scenario, in which poverty rates are projected to increase up to 2030 (see Appendix p. 22 for alternative scenarios). Three scenarios of alternative evolutions of BFP and BPC coverages are presented alongside this economic scenario: mitigation, baseline, and severe fiscal austerity. The figure also shows the projected under-5 mortality rates resulting from the combination of the economic and social protection scenarios from 2020 to 30. The mitigating scenario was associated with a continuous reduction in under-5 mortality rates. In contrast, the baseline austerity scenario would cause an increase and stabilization of mortality rates after the peak of the economic crisis, and the severe austerity scenario was associated with an expressive increase in mortality rates until 2030.

The projected mortality rates from the mitigation and baseline scenarios were compared by calculating the RR between their values for the year 2030. A similar exercise was performed between the mitigation and severe austerity scenarios. The RR between the mitigation and baseline scenarios was 0.761 (95% CI: 0.714–0.811), implying that the adoption of mitigation policies could avert 87,808 (95% CI: 73,096–102,661) under-five deaths compared to the baseline scenario over the period 2020–2030. Similarly, the RR between the mitigation and severe austerity scenarios was 0.65 (95% CI: 0.596–0.696), indicating that implementing mitigation measures instead of severe fiscal austerity could avert 148,736 (95% CI: 127,148–170,706) deaths over 2020–2030. The RR and numbers of averted deaths in alternative economic crisis scenarios, combined with these policy responses, are comparable in magnitude and are reported in the Appendix, p.22.

## Discussion

This study evaluated for the first time the combined effects of Social Pensions and Conditional Cash Transfers, which represent the backbone of Social Assistance, on reducing child and infant mortality over the past two decades in a large and unequal LMIC such as Brazil. Furthermore, using validated microsimulation models, we showed that expanding the coverage of both Social Pensions and Conditional Cash Transfers–in response to the current global economic crisis–could avert almost 150,000 under-five deaths by 2030, compared to fiscal austerity measures. To the best of our knowledge, this is the first study that performs a nationwide evaluation of the combined impact of SP and CCT on infant and child mortality over two decades in a LMIC, and uses these estimates to forecast their mitigation effect during an economic crisis and beyond.

A detailed description of the mechanisms that could explain the effects of each intervention on child health -as shown in - is provided in the Appendix, on P.3. Both programs provide money allowances to individuals of households with income per capita below an established threshold, which is adjusted periodically according to the inflation rate and the updated poverty line definitions (in 2019, the poverty line was U$ 47 for BPC and U$ 34 for BFP; and average money allowances transferred monthly was U$ 189 for BPC and U$ 36 for BFP). Cash transfer can improve the quantity and quality of the household food, and consequently the nutritional status of the children.^32^ On the other hand, the increased amount of available cash allows to buy hygiene and pharmaceutical products, which can have a direct effect on child health.^32^ Moreover, the benefits allow the mothers—often working in daily precarious jobs–to take days off work and take care of their sick children, if necessary, and to have time and economic resources to bring them to primary health care post or to the hospital.

The strong effect of the Social Pensions program—that targets elderly and disabled individuals–on child and infant mortality has never been shown in the literature and was somehow unexpected, but can be explained by several factors. Firstly, the BPC targets poor elderly and disabled individuals, usually living in extended vulnerable families and their children—whose health can benefit from the redistribution of the money allowances of the program.^33^ Second, a part of the disabled individuals beneficiaries of BPC are poor children in extremely vulnerable health conditions, and the receipt of economic support could significantly increase their survival chances. Additionally, the BPC transfers the minimum salary, which is about four times the basic value of the BFP.^11^ While some of the Rate Ratios associated with the BPC are higher then the ones of BFP, it is not possible to infer a stronger effect of one program on the other, due to the different and not comparable measures of coverage (BFP target population coverage versus BPC municipal coverage). It was not possible to estimate the BPC target population coverage due to the unavailability of data on the number of BPC eligible individuals for each municipality, which would have constituted the denominator of the target population coverage.

Regarding the Conditional Cash Transfer Program Bolsa Familia, while previous studies have shown that the BFP can reduce child mortality, in particular for poverty-related causes,^17^^,^^18^^,^^34^ none has systematically estimated its effects on infant, 1–4, and under-five mortality over the last two decades, and none has ever evaluated its impact according to municipality poverty levels. CCTs can improve child health and survival through two main mechanisms: the cash transfers, as described above, and the compliance with the health and education conditionalities (which are not present in BPC).^10^ BFP has been shown to increase vaccination coverage in children and reduce the number of mothers with no prenatal visits at the moment of delivery,^18^ while the remaining health conditionality, the periodic growth control of the children at the primary health care units, can approximate the mother and child to the healthcare system, allowing the identification of children with chronic or semi-sintomatic pathologies, besides children at risk of stunting and wasting.^32^ Moreover, the control of school attendance—promoted by BFP education conditionalities—could provide the children with basic health, nutrition and hygiene knowledge.^10^ The strongest effect of BFP target coverage in the poorest municipalities, with a gradient effect accorting to poverty levels, can be explained by the tendency of poverty-relief programs to be more effective in individuals and situations of higher vulnerability,^35^ and by the effects of BFP externalities^36^: in the poorest municipalities where BFP coverages are particularly high, even non-beneficiaries of the program could benefit from the greater money availability of the beneficiaries, which contribute to the reactivation of economic dynamics in the community. On the contrary, the BPC, which is less focused on extreme poverty, is not showing a clear gradient.

This study has some limitations. First, the selection of municipalities with a higher quality of vital information, which provided more reliable estimates but reduced their generalizability. However, while this kind of selection has been widely used in the previous studies,^16^^,^^18^^,^^20^ we have verified that the main findings were also sustained using all municipalities (Appendix p. 8). Second, the possibility of ecological fallacy, despite the fact that ecologic-level designs has been consistently used to assess the impact of CCT programs and other policy interventions, and that the use of smaller units of analysis such as the municipalities can mitigate this problem.^7^^,^^14^^,^^27^ Lastly, the current economic crisis scenario and future projections are extremely uncertain, and while recent reports show that our projections of poverty increase are reasonably conservative,^5^^,^^12^ we have tested a wide range of alternative economic crisis scenarios, showing that BFP and BPC have significant mitigation effects in different economic and poverty dynamics.

One of the main strengths of our study is the extensive sensitivity analyses performed, which confirmed the robustness of the findings, and all the triangulation analyses that demonstrate a high degree of confidence in the results of the impact evaluation, and confers robustness to the forecasted scenarios, which models have been internally and externally validated.

In conclusion, our study provides robust evidence of the role of Social Assistance, in particular of Social Pensions and Conditional Cash Transfers, in reducting child and infant mortality, and mitigating the impact of economic crises on child health. With the worsening of the current global multiple crises, and the increasing risk of a global recession, the survival of millions of vulnerable children is at risk, especially in LMIC. Our results underscore the importance of a preparedness and prompt response strategy based on the expansion of Social Assistance to minimize avoidable child deaths.

## Contributors

DR and DOR developed the study concept. GSM, JAO, TJA, and DC collected the data. DR, TJA, and JAO designed the study investigation. TJA, DC, and JAO did the data analysis. TJA, DC, DOR, and DR wrote the first draft of the manuscript. All authors contributed to data interpretation and reviewed and edited the manuscript. DR and DOR supervised the study process.

## Data sharing statement

All data used in this study are publicly available from the sources listed in the Appendix.

## Declaration of interests

**We declare no competing or conflict of interest.** However, we inform that TJA, DR, and DOR were funded by Bill & Melinda Gates Foundation, Grant Number: INV-027961; in partnership with Brazilian Ministry of Health, and Brazilian National Council for Scientific and Technological Development (CNPq)—call DECIT/CNPq [445743/2020-4]. TJA, JAO, DC, GSM, and DR were funded by Medical Research Council (MRC) UKRI, Grant_Number: MC_PC_MR/T023678/1.

## Appendix A Supplementary data

Web Appendix
