# Supplementary material for: The combined effect of social pensions and cash transfers on child mortality: evaluating the last two decades in Brazil and projecting their mitigating effect during the global economic crisis
Source: Lancet Reg Health Am. 2023 Nov 3;27:100618. doi: 10.1016/j.lana.2023.100618 (PMC10661114; doi:10.1016/j.lana.2023.100618)
Supplement: Web Appendix [file mmc1.docx]

**SUPPLEMENTARY WEBAPPENDIX**

**The combined effect of Social Pensions and Cash Transfers on child mortality: evaluating the last two decades in Brazil and projecting their mitigating effect during the global economic crisis**

**TABLE OF CONTENTS**

[PART I – SUMMARY OF SOCIAL PROGRAMS, DATA SOURCES AND GENERAL METHODOLOGY 2](#_Toc135397036)

[1. Background - social welfare state programs in Brazil 2](#_Toc135397037)

[*1.1.* *Bolsa Família Program (BFP)* 2](#_Toc135397038)

[*1.2.* *Benefício de Prestação Continuada (BPC)* 2](#_Toc135397039)

[*1.4. Mechanisms of the effect of BFP and BPC on health outcomes* 3](#_Toc135397040)

[2. Dataset 3](#_Toc135397041)

[*2.1.* *Data sources* 3](#_Toc135397042)

[*2.2.* *Interpolation and extrapolation method* 4](#_Toc135397043)

[*2.2.1.* *Discussion and Results* 5](#_Toc135397044)

[PART II – RETROSPECTIVE ANALYSIS 5](#_Toc135397045)

[3. Empirical methods 5](#_Toc135397046)

[*3.1.* *Negative binomial regression – Fixed Effects* 5](#_Toc135397047)

[4. Results 6](#_Toc135397048)

[*4.1.* *Descriptive Analysis* 6](#_Toc135397049)

[*4.2.* *Triangulation – Difference-in-difference with propensity score matching* 9](#_Toc135397051)

[*4.3.* *Sensitivity Analyses* 10](#_Toc135397052)

[PART III – FORECASTING ANALYSIS 22](#_Toc135397053)

[5. Description of the forecasting methodology 22](#_Toc135397054)

[6. Purpose of the forecasting and its applications 22](#_Toc135397055)

[7. Inputs, outputs, and other parameters 22](#_Toc135397056)

[*7.1. Scenarios of poverty and coverage of social welfare programs* 22](#_Toc135397057)

[8. Prediction methodology 24](#_Toc135397058)

[*8.1. External validation* 24](#_Toc135397059)

[9. Sensitivity analysis 25](#_Toc135397060)

[10. Main limitations 26](#_Toc135397061)

[REFERENCES 27](#_Toc135397062)

# PART I – SUMMARY OF SOCIAL PROGRAMS, DATA SOURCES AND GENERAL METHODOLOGY

# Background - social welfare state programs in Brazil

## ***Bolsa Família Program (BFP)***

The Bolsa Família is the Brazilian Conditional Cash Transference (CCT) program that aims to attenuate the effects of poverty through a minimum cash transfer for beneficiary families, and to break the intergenerational cycle of poverty through investment in education and health conditionalities.^1^ This important socio-economic intervention was established in 2004 by Law № 10,836, of January 9, 2004, with the last monetary restatement modified by Decree № 9,396, of May 30, 2018, in which households are eligible for the program if their per capita income is equal to or less than R$89.00 (approximately US$19 at current 2022 prices) or if they are poor families with income up to R$ 178.00 (approximately US$ 38 at current 2022 prices) and one member is a child up to 17 years old or a pregnant woman (or a woman who just gave birth).^2^

In the health area, conditionalities concern the monitoring of vaccination and nutritional surveillance of children, as well as prenatal care of pregnant women and the puerperium, and should be attended at the ESF units. Some studies have shown the effect of BFP on child morbidity and mortality1, and on other health outcomes associated with poverty, such as malnutrition, diarrhoea, lower respiratory infections, and vaccine preventable diseases.^1^

The BFP is one of the largest CCT in the world with more than 13.9 million families benefiting throughout Brazil, and was recently remodeled and called Axílio Brasil Program (ABP) by Law № 14,284, of December 29, 2021. Basically, this new program maintains the conditionalities and general structure of the BFP, but it increases the coverage and the value transferred through changes in the criteria of poverty and extreme poverty; where families earning up to R$105.00 per capita (approximately US$22 at current 2022 prices) and up to R$210.00 (almost US$45 at current 2022 prices) are considered extremely poor and poor, respectively. No studies were found that directly evaluate the ABP, however studies that simulated future scenarios of increased coverage of BFP show improvements in health outcomes.^3^

## ***Benefício de Prestação Continuada (BPC)***

The Conditional Welfare Benefit (BPC) is a non-contributory social protection program that was implemented by the 1988 Brazilian Federal Constitution to ensure the welfare of the elderly (above age 65) and disabled population of all ages, included Childs. The program guarantees a minimum wage payment to the target population whose family income per capita does is below a quarter of the minimum salary. The objective is to ensure the subsistence and independence, and also guarantee the reduction of poverty and vulnerability of the elderly and disabled population. By May 2020, about 2.08 and 2.57 million of the elderly and disabled population are assisted by the BPC program^1^. The government bodies responsible for the management and operationalization of the BPC program are the Ministry of Citizenship (previously called “Ministry of Social Development and Fight Against Hunger” – MDS), National Social Assistance Secretariat (SNAS), Ministry of Social Security (MPS), National Social Security Institute (INSS), and the Social Assistance Reference centers (CRAS).

The BPC program encompasses two important social welfare initiatives designed for the disabled beneficiaries: the BPC School Program and the BPC Employment Program. The BPC School Program aims to enable access and follow-up the school attendance record of the disabled beneficiaries below the age of 18. The BPC Employment Program helps the beneficiaries between age 16 and 64 who desire to work but encounter barriers to gain access into the labor force through vocational trainings and educational courses. The channel thorough with this initiative is executes is the Program for Access to Technical Education and Employment (PRONATEC) managed by the Ministry of Education.

The participation in the BPC program is frequently (every two years) reassessed to ensure that the beneficiaries continue to meet the eligibility condition regarding family income, and also occasionally regarding the deficiency status, i.e., deficient beneficiaries who recovered from the reported illness and have sufficient income are suspended are eliminated from the program. Moreover, the benefits from the BPC are exclusive to the beneficiary and not transferrable.

## ***1.4. Mechanisms of the effect of BFP and BPC on health outcomes***

There are several mechanisms through which the BFP program affect health outcomes. First, the BFP conditions beneficiaries to a minimum usage of health services for child and maternal health, i.e., the conditionality effect.^4,5^ Second, the income transferred to poor and extremely poor families improves the nutrition and living conditions of these families, i.e, the income effect^6^. Third, long-term exposure to the health conditionality of the BFP promote behavior changes and adherence of beneficiary families towards health care^7^.

Previous studies show that the BPC affect health outcomes through increased access to healthcare and medications^7,8^; better economic conditions, i.e., reduced poverty^9^, and; improved nutrition^10^. Regarding the strong mitigating effect of the BPC on under-5 mortality, studies have found evidence of improved health outcomes of children living in the same households with social pension beneficiaries.^11–13^ It is also important to recall that the BPC attends to both the elderly population and deficient population irrespective of age.

**Figure 1: Mechanisms linking the Social Pension Program and Bolsa Familia Program to health outcomes.**

**
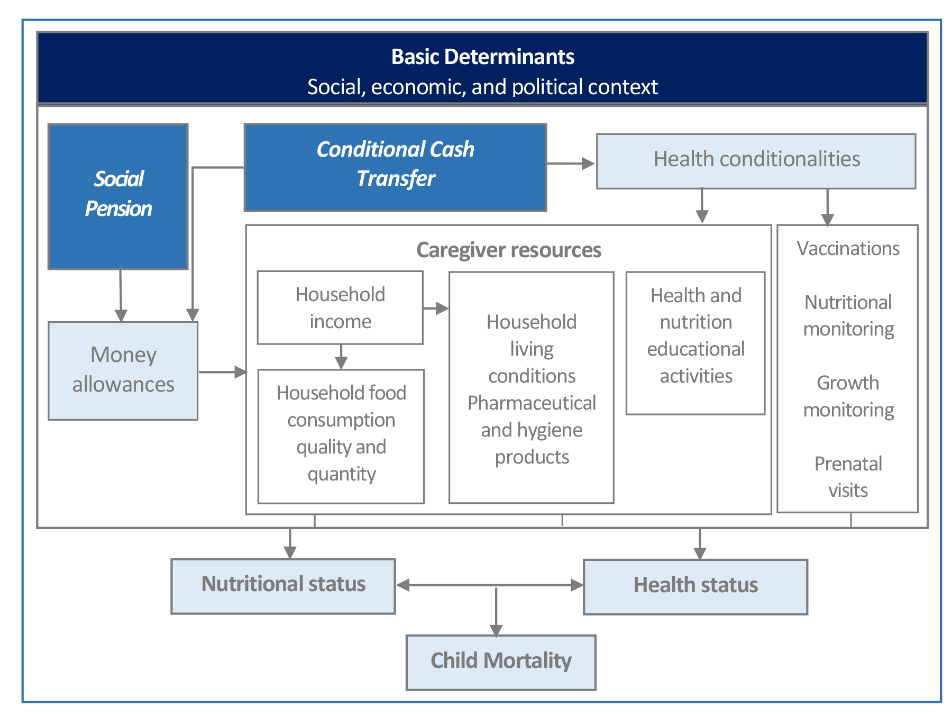
**

# Dataset

## ***Data sources***

The data used in this study were obtained from various governmental platforms detailed in Web Table 1. All the variables used in this study are aggregated to the municipal level. However, the data for some variables were not available for specific years and municipalities, therefore we performed linear interpolation as detailed in Section 2.2. of this supplementary document.

**Web Table 1: Sources and description**

| **Variable** | **Years** | **Units of analysis** | **Source** | **Link** |
| --- | --- | --- | --- | --- |
| Mortality and Morbidity | 2000 to 2019 | Municipality | DATASUS - SIM | <https://datasus.saude.gov.br/mortalidade-desde-1996-pela-cid-10> |
| Population estimates | 2000 to 2019 | Municipality | IBGE – Census | <https://www.ibge.gov.br/> |
| Municipal population age sex-race distributions | 2000 to 2019 | Municipality | IBGE – Census | <https://www.ibge.gov.br/> |
| Livebirth | 2000 to 2019 | Municipality | DATASUS - SINASC | <https://datasus.saude.gov.br/nascidos-vivos-desde-1994> |
| Gini index | 2000 and 2010, 2001-2019 | State and Municipality | IBGE - Census; IBGE - PNAD; and IBGE - PNADC | <https://sidra.ibge.gov.br/pesquisa/censo-demografico/demografico-2010/inicial> |
| Illiteracy rate (Percentual of people older than 15 years who are illiterate) | 2000 and 2010, 2001-2019 | State and Municipality | IBGE - Census; IBGE - PNAD; and IBGE - PNADC | <https://sidra.ibge.gov.br/pesquisa/pnadct/tabelas> |
| Poverty rate (Poor population estimate) | 2000 and 2010, 2001-2019 | State and Municipality | IBGE - Census; IBGE - PNAD; and IBGE - PNADC | <https://www.ibge.gov.br/estatisticas/sociais/populacao/9127-pesquisa-nacional-por-amostra-de-domicilios.html?=&t=downloads> |
| Percentual of household with piped water | 2000 and 2010, 2001-2019 | State and Municipality | IBGE - Census; IBGE - PNAD; and IBGE - PNADC | <https://sidra.ibge.gov.br/pesquisa/censo-demografico/demografico-2010/inicial> |
| Percentual of household with sewage/sanitation | 2000 and 2010, 2001-2019 | State and Municipality | IBGE - Census; IBGE - PNAD; and IBGE - PNADC | <https://sidra.ibge.gov.br/pesquisa/pnadct/tabelas> |
| Bolsa Família program coverage | 2004 to 2019 | Municipality | MDS | <https://aplicacoes.mds.gov.br/sagi/vis/data3/data-explorer.php> |
| Benefício de Prestação Continuada Program coverage (Brazilian non-contributory pension benefit) | 2004 to 2019 | Municipality | MDS | <https://aplicacoes.mds.gov.br/sagi/vis/data3/data-explorer.php> |
| Hospital bed rate (beds per 1,000 people) | 2000 to 2019 | Municipality | DATASUS - CNES | <https://datasus.saude.gov.br/cnes-recursos-fisicos> |
| Doctor rate (Physicians per 1,000 people) | 2000 to 2019 | Municipality | DATASUS - CNES | <https://datasus.saude.gov.br/cnes-equipes-de-saude> |

**Note**: DATASUS - Department of Informatics of the Unified Health System (Departamento de Informática do Sistema Único de Saúde); SIM - Mortality Information System (Sistema de Informações sobre Mortalidade); DAB - Department of Primary Care (Departamento de Atenção Básica); CNES - National Register of Health Establishments (Cadastro Nacional de Estabelecimentos de Saúde; SINACS - Live Birth Information System (Sistema de Informações sobre Nascidos Vivos); IBGE - The Brazilian Institute of Geography and Statistics (Instituto Brasileiro de Geografia e Estatística); MDS - The Ministry of Social Development and Fight against Hunger (Ministério do Desenvolvimento Social e Combate à Fome); PNAD - (Pesquisa Nacional por Amostra de Domicílios); PNADC - Continuous PNAD Quarterly (Pesquisa Nacional por Amostra de Domicílios Contínua).

## ***Interpolation and extrapolation method***

Longitudinal data collection regarding socioeconomic variables is not frequent at the municipal level given the high cost of conducting surveys in Brazil. Therefore, in most cases empirical studies resort to the linear interpolation of data; settle with the use of census cross-sectional data, or; use state-level data. In Brazil, the most robust municipal-level socio-economic data are obtained from the national census, which was last conducted in the year 2000 and 2010. However, the Brazilian National Household Sample Survey (PNAD), in which the smallest unit of aggregation is the state, is conducted every year.

In this study, instead of performing conventional linear interpolation of the two data points (2000 and 2010 for each municipality, we perform a more robust form of linear interpolation whereby, in addition to the linear interpolated data, we input variation extracted from state-level data. This procedure can be simply understood as the linear interpolation of municipal data adjusted by state-level information.

This procedure is carried out in four steps:

1. Given the complete state-level information, the first step is then to estimate an Ordinary Least Squares (OLS) linear regression of each variable of interest as a function of time $t=2000,\ldots,2018$. Then, we obtain the residuals $e_{t}$, as follows:

$\hat{e}_{jt} =y_{jt}-z_{jt} ,$ where $z_{t}= \hat{\beta_{0j}}+ \hat{\beta_{1j}t}$ (1),

with $t = 2000, \ldots, 2019$ and $\hat{\beta_{0it}}, \hat{\beta_{1it}}$ being the regression coefficients estimated by the OLS method. Therefore, 20 residuals will be obtained for each state, corresponding to the 2000-2019 period.

1. For the municipality level, using the census information available (years 2001 and 2010), the data was inter-extrapolated following the next sub-steps,
2. We calculate the linear equation between the two data points $(2000, y_{ij2000})$ and $(2010,y_{ij2010})$, where $y_{ijt}$ denotes the value of $y$ at the i-th municipality from the j-th state at the time $t$. This equation will be of the form,

$$z_{ijt}= \beta_{0ij}+ \beta_{1ij}t (2)$$

3. Subsequently, we calculate the $z_{ijt}$ values for $t = 2001,\ldots, 2009,\ldots,2011,\ldots, 2018$ using equation (2) (the values corresponding to t = 2000 and t= 2010 are already available from the census, so they do not need to be imputed).
4. Finally, we impute $y_{ijt}$ for $t \neq2000, 2010$ as:

$y_{ijt} = z_{ijt} + e_{it} (3)$

where $e_{it}$ corresponds to the state residual calculated from the regression in (1).

## ***Discussion and Results***

The proposed method imputes the state behavior observed in PNAD microdata to annual municipal fluctuations in non-census periods, that is, 2001 to 2009, and 2011 to 2019. With this it is possible to capture important effects that occurred in this period, such as breaks in trends and temporal shocks caused by the Brazilian economic crises of 2008, 2013 and 2015^3^; and its consequences on several socioeconomic variables, such as the increase in income inequality and poverty of families in Brazil^14^.

A total of 892,134 values were generated for 9 socioeconomic variables, in 5,507 municipalities over 18 years with this method. The interpolated variables were Illiteracy rate, urbanization rate, household infrastructure (garbage, sewage, piped water), and inequality and income variables (family income, Gini index, poverty rate and extreme poverty rate). Some of these variables were used as control variables for the models, none of the outcome variables (mortality and morbidities) or exposure variables (BPC and BFP) were interpolated. In the end, it was observed that the interpolated variables improved the control and precision of the retrospective and predictive models.

# PART II – RETROSPECTIVE ANALYSIS

# Empirical methods

## *Negative binomial regression – Fixed Effects*

We estimate Fixed Effect models using the negative binomial method to retrospectively evaluate and forecast the impact of each welfare social policy on health outcomes. The equation which describes the linear relationship between the health outcomes (mortality rate) and covariates is given by:

$$Log \mu_{it} = \alpha_{i} +\sum_{q=1}^{3} \beta_{q}BFP_{qit}+\sum_{s=6}^{7} \beta_{s}BPC_{sit}+ \beta_{8}T_{2008} +$$

$$\beta_{9}T_{2013} + \beta_{10}T_{2015}+\sum_{k=11}^{16} \beta_{k}X_{kit},$$

where:

$t$ refers to the year, $i$ refers to an individual municipality, and $q,r,s$ are indexes representing the categories of each intervention,

$\mu_{it}$ are the mean of the welfare state variables (mortality rate for age group) observed at the municipality $i$ in year $t$,

$BFP_{qit}$ are the dummies representing the BFP coverage categories ($4$ groups) observed at the municipality $i$ in year $t$ with a coefficient of $\beta_{q}$,

$BPC_{sit}$ are the dummies representing the BPC coverage categories (terciles) observed at the municipality $i$ in year $t$ with a coefficient of $\beta_{s}$,

$T_{2008}, T_{2013}, T_{2015},$ are dummy variables representing previous crisis events with coefficients $\beta_{8}, \beta_{9}, \beta_{10}$ respectively,

$X_{kit}$ represents different control covariates, each one with a coefficient of $\beta_{k}$ (Poverty, illiteracy, fertility and doctors rates and percentage of households with garbage collection).

$\alpha_{i}$ is the fixed effect (time-invariant) term for each municipality.

This formula above is a log-linear representation of the Poisson distribution, which can be represented by:

| $Pr\left( Y_{i}=y_{i} \right)=\frac{e^{-\mu_{i}}\left( \mu_{i} \right)^{y_{i}}}{y_{i}!}, with y=0,1,2\ldots,n$ and ${log \mu}_{i}= \beta_{1}X_{1i}+\beta_{1}X_{2i}+\ldots+\beta_{k}X_{ki}$ | [1] |
| --- | --- |

where:

$Pr$ is the probability of $y$ events (U5 death count). The parameter $\mu$ is the mean incidence rate of y (under-5 death count) per unit of exposure t (livebirths). The parameters $\beta_{1},\ldots\beta_{k}$ are unknown parameters that are estimated from the data set described in section 2 of this web appendix, for the X control variables.

The negative binomial regression can be obtained by including, in the Poisson regression model [1], a gamma noise variable, $\alpha$(which has a mean of 1 and a scale parameter of $\nu$).

$$Pr\left( Y=y_{i}|\mu_{i},\alpha\right)=\frac{\Gamma\left( y_{i}+\alpha^{-1} \right)}{\Gamma\left( y_{i}+1 \right)\Gamma\left( \alpha^{-1} \right)}\left( \frac{1}{1+{\alpha\mu}_{i}} \right)^{\alpha^{-1}}\left( \frac{\alpha\mu_{i}}{1+{\alpha\mu}_{i}} \right)^{y_{i}}$$

where:

The parameter μ is the mean incidence rate of y (under-5 death count) per unit of exposure (livebirths), and Γ(.) is the gamma function.

# Results

## *Descriptive Analysis*

In this section (from Web Figures 1 – 3), we present figures that describe the temporal dynamics of the BFP and BPC, alongside the health outcomes (Under 5 mortality rates by age group and overall) from the year 2004 to 2019. During this period, the overall rate of U5 mortality reduced expressively, except for after the 2015 crisis, especially for the under-5 subgroups.

Regarding social welfare programs, Web Figures 4-5, showed that the coverages of the BFP and BPC increased steadily from 2004 to 2018, although at a diminishing rate due to the long-term fiscal policy measure adopted since the 2013 economic crisis. In the year 2019, the reduction of the coverages of these programs was clear. Web Figure 6 shows the average of mortality rate over the BFP and BPC coverage levels (in increasing order). In most cases, an inverse or negative correlation is perceptible between coverage levels and health outcomes.

**Web Figure 1: Child mortality rate (under 5 age group), 2004-2019.**


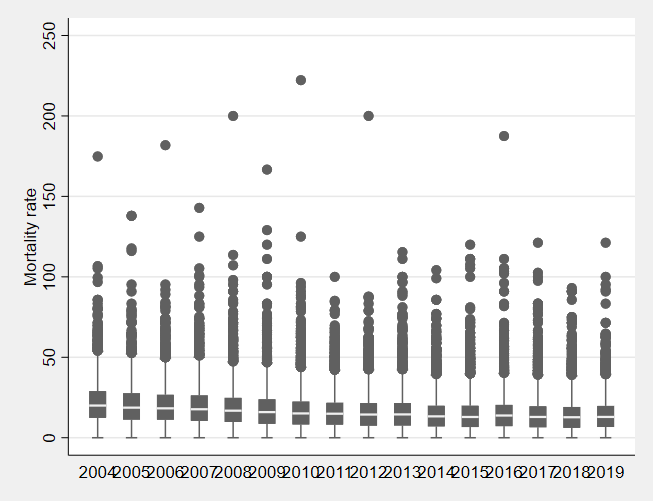

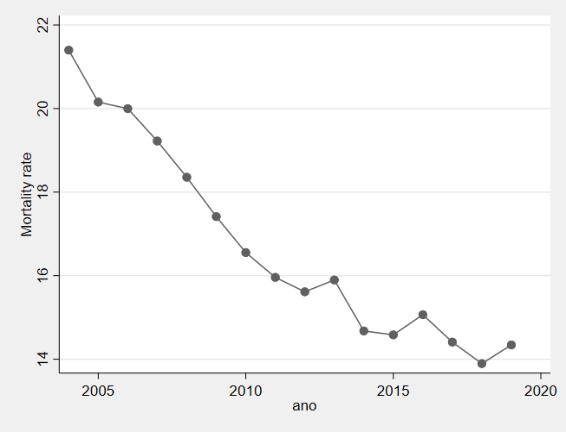


**Note:** Data are in Under-5 mortality rate per 1,000 livebirths (y-axis), in a 2004 to 2019 period (x-axis).

**Web Figure 2: Infant mortality rate (under 1 age group), 2004-2019**

| 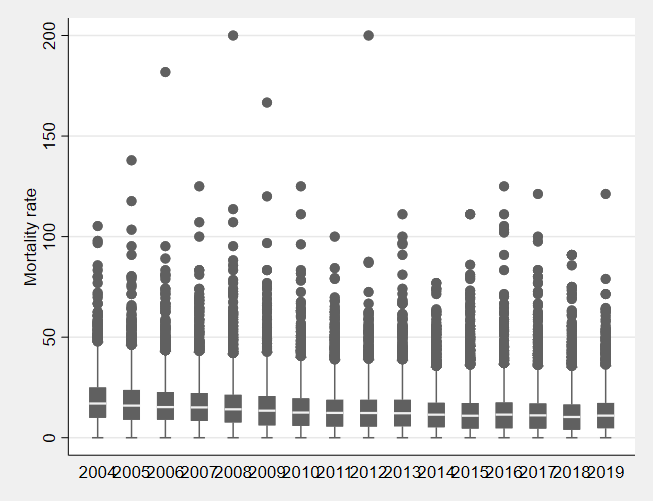 | 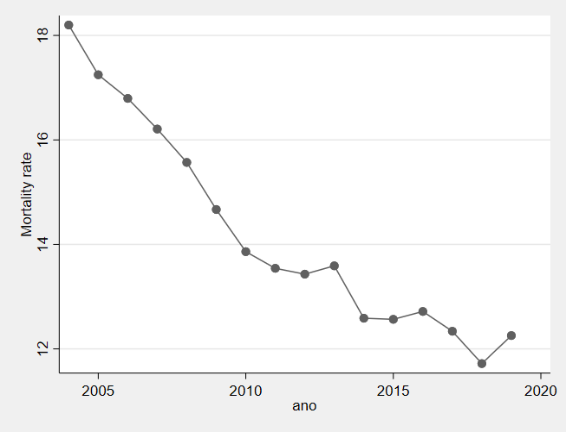 |
| --- | --- |

**Note:** Data are in Under-1 mortality rate per 1,000 livebirths (y-axis), in a 2004 to 2019 period (x-axis).

**Web Figure 3: Toddler mortality rate (1 to 4 age group), 2004-2019**

| 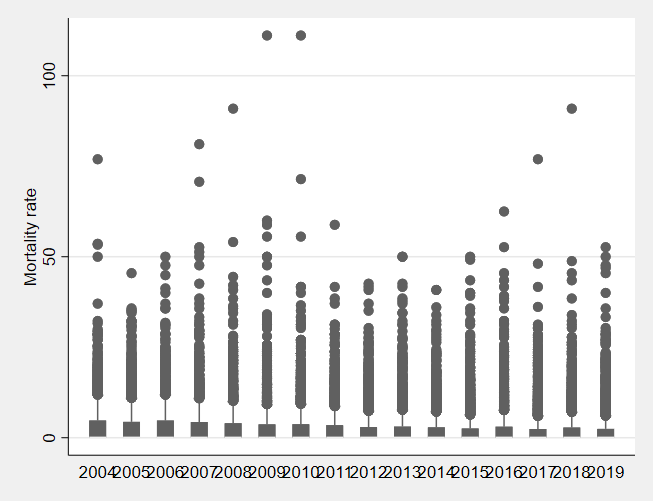 | 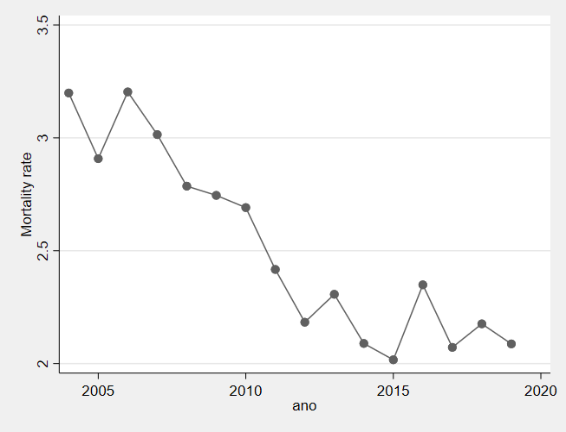 |
| --- | --- |

**Note:** Data are in Toddler mortality rate (1 to 4 years) per 1,000 livebirths (y-axis), in a 2004 to 2019 period (x-axis).

**Web Figure 4**: Coverage of the Bolsa Família Program (target population), 2004-2019.


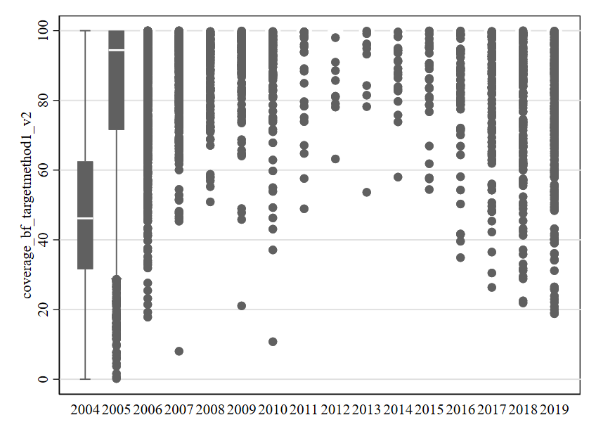

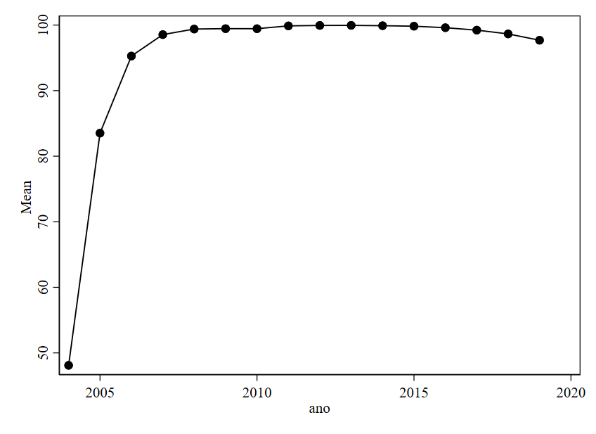


**Note:** Data are in Bolsa Familia Program coverage – determined by dividing the number of families enrolled in the program by the eligible population (y-axis) – in a 2004 to 2019 period (x-axis).

**Web Figure 5: Coverage of the BPC Program (population), 2004-2019.**


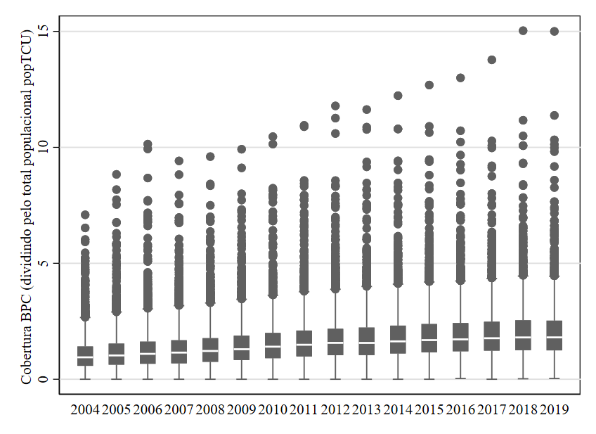

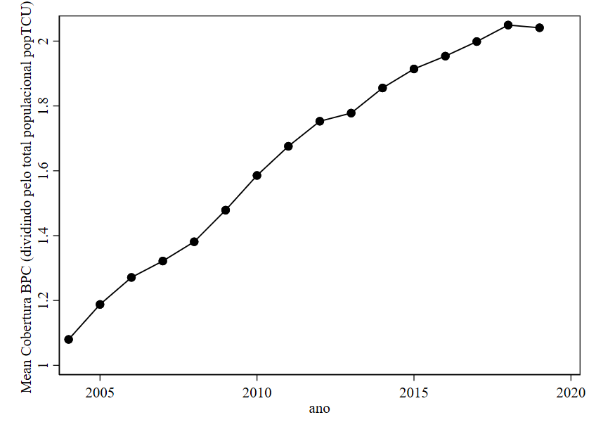


**Note:** Data are in Benefício de Prestação Continuada (BPC) Program coverage – determined by dividing the number of families enrolled in the program by all population of each municipality (y-axis) – in a 2004 to 2019 period (x-axis).

**Web Figure 6: Average mortality rate by the level of coverage of the BFP and BPC social welfare programs, 2004-2019.**

| **BFP Program on Mortality** | **BPC program on Mortality** |
| --- | --- |
| 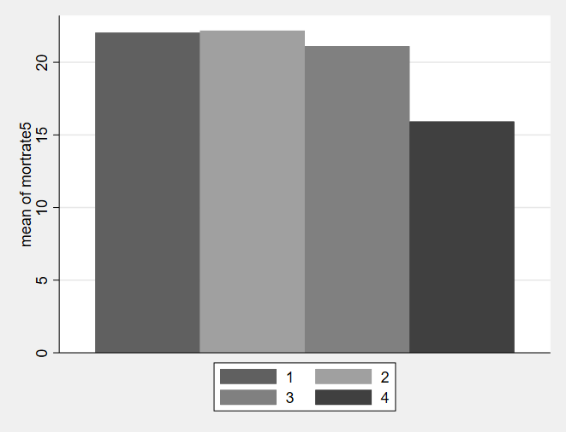 | 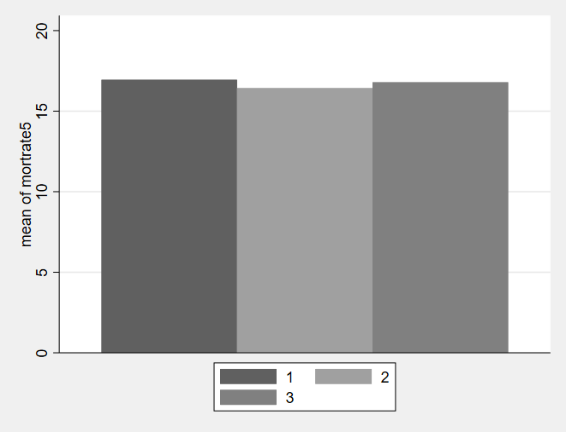 |

**Web Figure 7: Average coverage of the BFP and BPC, by municipality**

| **BFP target coverage (2004)** | **BPC coverage (2004)** |
| --- | --- |
| 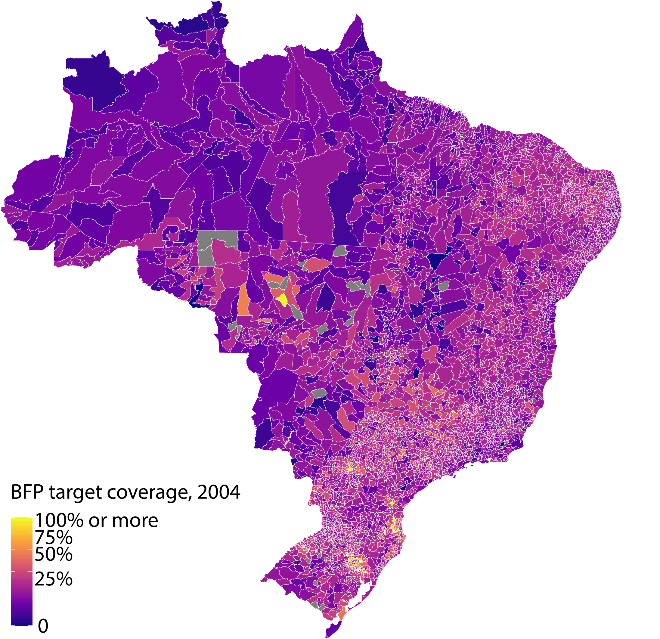 | 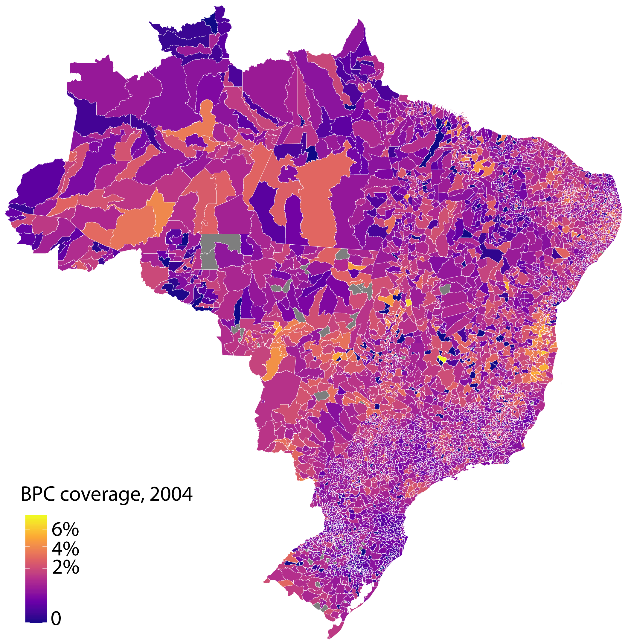 |
| **BFP target coverage (2019)** | **BPC coverage (2019)** |
| 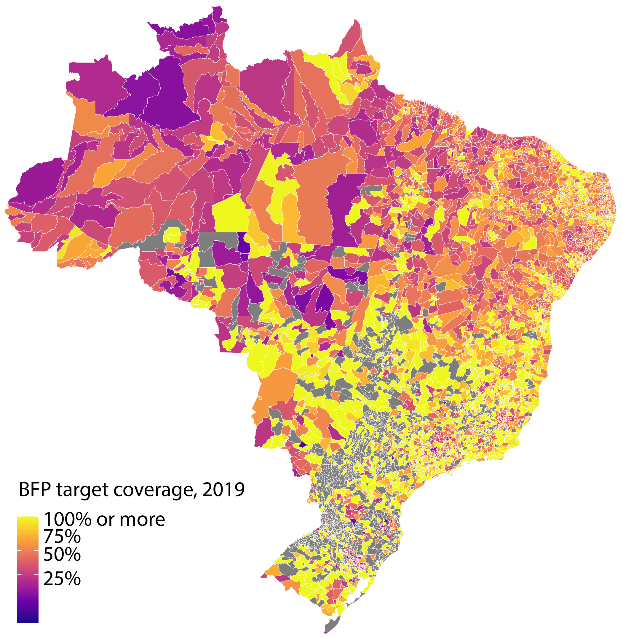 | 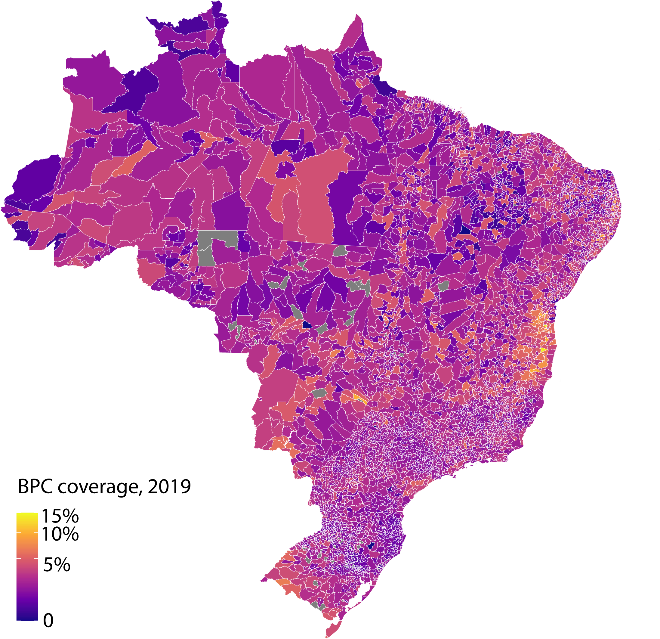 |

## *Triangulation – Difference-in-difference with propensity score matching*

We analyzed the effect of BFP and BPC programs on child mortality (U5MR) by difference-in-difference (DID) with propensity score matching (PSM) as a triangulation approach.^15^ For the Bolsa Familia Program, we used 602 municipalities with low coverage (BFP<=29.9%) and compared them with 1,944 municipalities with high coverage (BFP>30%) in the years 2004 and 2019. We used the same strategy for the social pension program, using 485 municipalities with low coverage (BPC<=0-32.9 percentile) and compared them with 1,231 municipalities with high BPC coverage (BPC>33-100 percentile) in the years 2004 and 2019. Thus, municipalities with low coverage of each of these programs receive a value equal to 0 (control) and municipalities with medium to high coverage receive a value equal to 1 (treated).

We also tested three different ways of estimating the DID: the first and second approaches we used the "diff" command in STATA, one with pure DID model and other adding the Propensity Score Matching before the DID model. On the third approach, we used the step-by-step procedure described in the World Bank handbook,^16^ which allows estimating DID with a negative binomial panel of fixed effects and with coefficients in Rate Ratio (RR) . We prefer this third one because it allows a more direct comparison with the results reported in the manuscript. The table below further separates these analysis strategies.

**Web Table 2. Different strategies for difference in difference models**

| **Analysis strategies** | **Control (dummy=0)** | **Treated (dummy=1)** | **Time** | **Table** |
| --- | --- | --- | --- | --- |
| BFP | Low BFP coverage (<30%) | Intermediate to high BFP coverage (>=30%) | 2004 and 2019 | Table 3 (“diff”) – only DID model and DID with PSM;  Table 4 – DID with IRR fixed effect negative binomial models |
| BPC | Low BPC coverage  (<33 tercile) | Intermediate to high BPC coverage  (>33 tercile) | 2004 and 2019 |

Web-Tables 3 and 4 show the result of DID with PSM, coverages of BFP and BPC programs were associated with a statistically significant reduction in child mortality rates, with incidence-rate ratios(IRR) of 0·990(95%CI:0·987-0·992). Even after approaching the municipalities by characteristics observed by the kernel matching method, the first difference in 2004 shows that municipalities with some coverage of the CCT program had a higher infant mortality rate compared to municipalities without coverage. The second difference, in 2019, shows that both municipalities with 0% CCT coverage and those with some level of CCT coverage reduced their child mortality. However, municipalities with coverage of CCT programs had an even greater reduction, so that the difference in difference was statistically significant. Thus, the CCT programs contributed to the reduction of infant mortality, being compatible with the results already found and described in the main manuscript, so that these results by DID with PSM reinforce the results found by the fixed effect panel with negative binomial, being a form of triangulation of results.

**Web Table 3**. **Difference-in-difference with Propensity Score Matching for the association between child mortality rates and intermediate to high Conditional Cash Transference (CCT) coverage, in 2004 and 2019.**

|  | **BFP** | | **BPC** | |
| --- | --- | --- | --- | --- |
|  | **DID** | **DID WITH PSM** | **DID** | **DID WITH PSM** |
|  | **Child**  Under 5 years | **Child**  Under 5 years | **Child**  Under 5 years | **Child**  Under 5 years |
| Before (2004) |  |  |  |  |
| Control | 17.016 | 16.611 | 18.958 | 18.929 |
| Treated | 16.436 | 16.436 | 18.630 | 18.630 |
| 1^st^ Difference (T-C) | -0.581***  (0.111) | -0.176**  (0.055) | -0.328***  (0.125) | -0.300**  (0.122) |
| After (2019) |  |  |  |  |
| Control | 15.514 | 15.439 | 19.215 | 19.167 |
| Treated | 15.045 | 15.045 | 18.224 | 18.224 |
| 2^nd^ Difference (T-C) | -0.469***  (0.111) | -0.394***  (0.000) | -0.991***  (0.123) | -0.944***  (0.205) |
| **Diff-in-Diff** | **0.112**  **(0.158)** | **-0·218***  **(0·129)** | **-0.663*****  **(0.175)** | **-0.644*****  **(0.238)** |
| Number of observations |  |  |  |  |
| Control | 1,204 | 1,204 | 963 | 963 |
| Treated | 3,888 | 3,888 | 2,445 | 2,445 |
| Total | 5,092 | 5,092 | 3,408 | 3,408 |
|  |  |  |  |  |
| Number of municipalities |  |  |  |  |
| Control | 602 | 602 | 485 | 485 |
| Treated | 1,944 | 1,944 | 1,231 | 1,231 |
| Total | 2,546 | 2,546 | 1,716 | 1,716 |

**Source:** Author's data analysis for 5,091 observations – 2,546 municipalities in Brazil, the years 2004 and 2019.

**Note:** Data are in mortality rate per 1,000 livebirths, with standard errors in parentheses. The symbols ‘***’ , ‘**’ and ‘*’ denote significance at 1%, 5%, and 10% respectively. We use the STATA "diff" command, with kernel matching (PSM) to approximate the compared municipalities according to their observable characteristics.

**Web Table 4.** **Incidence-Rate Ratios from the difference-in-difference fixed effect negative binomial models by under 5 age group for the association between mortality rates and intermediate to high Conditional Cash Transference (CCT) coverage, in 2004 and 2019.**

|  | **Under-5 Mortality Rate** | | |
| --- | --- | --- | --- |
|  |  | **Bolsa Familia Program (BFP)** | **Benefício de Prestação Continuada (BPC) Program** |
| **CCT target coverage (dummy)** |  | 5.62864e+12*** |  |
| - |  | [2.060e+08-1.538e+17] |  |
|  |  | 0.986*** |  |
| Intermediate to high (>=30%) |  | [0.982-0.991] |  |
| **BPC coverage (dummy)** |  |  | 732718032.9*** |
| - |  |  | [72873.173-7.367e+12] |
|  |  |  | 0.990*** |
| Intermediate to high (>33 tercile) |  |  | [0.986-0.995] |
| Control variables |  |  |  |
|  |  | Yes*** | Yes*** |
| **Number of observations** |  | **5,057** | **3,408** |
| **Number of municipalities** |  | **2,546** | **1,716** |

**Source:** Author's data analysis for 5,057 observations – 2,546 municipalities in Brazil, in 2004 and 2019 period.

**Note:** Data are in Rate Ratio (RR) coefficients (95% CI) unless otherwise specified. The confidence intervals are in brackets. The symbols ‘***’ , ‘**’ and ‘*’ denote significance at 1%, 5%, and 10% respectively.

## ***Sensitivity Analyses***

Concerning our study about the effect of social protection on child mortality, we developed several sensitivity tests in order to guarantee the robustness of our results.

As a first test, for choosing between a random and a fixed effect, we performed the Hausmann test for the child mortality data. By following different goodness of fit criteria including AIC, BIC and the Log-likelihood, we conclude that the fixed effects model were more adequate to analyze the effects of cash transfer and social pension policies on this kind of mortality.

Secondly, we fit the same models for the same age groups without filtering the data for adequate information (using the 5,507 municipalities), in order to see if our results hold.

As observed in Web Table 5, the direction and the significance of the intervention effects are preserved for most of the age groups. The gradient structure of the interventions is also preserved, indicating that the mitigation effects of this programs increase as the coverage increases.

Thirdly, we assess the period delimitation from 2004 to 2019 by using the data from 2000 to 2019 and considering that the PBF was only implemented since 2004. Following Web Table 6, we concluded that the results from both periods hold.

As a fourth test, we decided to include additional covariates to look for additional effects. Since similar control variables had been already included into the models, we decided not to include the new ones into the analysis. As shown in Web Table 7, the cumulative addition of these new redundant variables did not affect the previous results, proving that the estimates are stable.

Fifth, we also estimated models considering all covariates continuous. Web Table 8 shows that the effect of PBF and BPC are still aligned with previous results using dichotomized variables.

Sixth, since PBF and BPC are not just correlated but conditioned to poverty, we estimate the overall models for child mortality, with and without the covariate representing this characteristic for different municipalities. Web Table 9 allowed us to see that there is little or not effect of collinearity between poverty and these interventions.

Seventh, we tested four additional models with zero and different time shock controls (Web Table 10) and compared the results to our final overall models. Nevertheless, the overall results continue stable.

Eight, through different criteria including the Log – likelihood, AIC and BIC (See Web Table 11), we compared the Negative binomial and the Poisson regression models concluding that the first one was more adequate for this data. This is reasonable since the negative binomial regression takes the data overdispersion into account, compared to the Poisson which does not.

Ninth, in Web Table 12, we tested the heterogeneity of effects between poverty quartiles in each of the infant mortality subgroups. It was observed that the effects of the BFP and BPC programs are related to the highest quartiles and have no effect on the lowest quartiles, which reinforces the relationship between these programs and the poorest municipalities.

Finally, we estimate the model by adding binary controls for the regions of Brazil (see Web Table 13). Once again we observe that the results remain robust even with this specification change.

**Web Table 5– Empirical results for child mortality models without filter for adequate information – for all municipalities, 2004-2019, Brazil**.

|  | **Under 1** | | **From 1 to 4** | | **Under 5** | |
| --- | --- | --- | --- | --- | --- | --- |
|  | **Without controls** | **Wth controls** | **Crude** | **Adjusted** | **Crude** | **Adjusted** |
| BFP target coverage |  |  |  |  |  |  |
| Low (0-30%) | 1 | 1 | 1 | 1 | 1 | 1 |
|  | (1·000-1·000) | (1·000-1·000) | (1·000-1·000) | (1·000-1·000) | (1·000-1·000) | (1·000-1·000) |
| Intermediate (30-70%) | 1·002 | 1·007 | 1·012 | 1·020 | 1·004 | 1·009 |
|  | (0·980-1·025) | (0·985-1·030) | (0·964-1·063) | (0·972-1·071) | (0·983-1·025) | (0·988-1·030) |
| High (70-100%) | 0·950*** | 0·968*** | 0·932*** | 0·958* | 0·948*** | 0·967*** |
|  | (0·929-0·971) | (0·947-0·990) | (0·888-0·978) | (0·912-1·005) | (0·928-0·968) | (0·947-0·987) |
| Consolidated (≥100%) | 0·843*** | 0·893*** | 0·824*** | 0·890*** | 0·841*** | 0·893*** |
|  | (0·826-0·861) | (0·874-0·912) | (0·787-0·863) | (0·849-0·933) | (0·825-0·858) | (0·875-0·910) |
| BPC municipal coverage |  |  |  |  |  |  |
| Low (0-33%) | 1 | 1 | 1 | 1 | 1 | 1 |
|  | (1·000-1·000) | (1·000-1·000) | (1·000-1·000) | (1·000-1·000) | (1·000-1·000) | (1·000-1·000) |
| Intermediate (33-66%) | 0·911*** | 0·922*** | 0·894*** | 0·908*** | 0·908*** | 0·920*** |
|  | (0·902-0·920) | (0·913-0·931) | (0·874-0·915) | (0·887-0·929) | (0·900-0·916) | (0·911-0·928) |
| Consolidated (66-100%) | 0·803*** | 0·847*** | 0·767*** | 0·823*** | 0·797*** | 0·844*** |
|  | (0·792-0·814) | (0·836-0·859) | (0·743-0·791) | (0·797-0·850) | (0·787-0·807) | (0·833-0·855) |
| Proportion of individuals older than 15 years who are illiterate (%) |  | 1·020** |  | 1·058*** |  | 1·026*** |
|  |  | (1·004-1·036) |  | (1·022-1·096) |  | (1·011-1·041) |
| Poverty rate (%) |  | 1·059*** |  | 1·081*** |  | 1·061*** |
|  |  | (1·046-1·073) |  | (1·050-1·113) |  | (1·049-1·074) |
| Urbanization rate (%) |  | 0·990 |  | 0·969 |  | 0·986 |
|  |  | (0·969-1·011) |  | (0·925-1·015) |  | (0·967-1·006) |
| Fertility rate (%) |  | 1·079*** |  | 1·037** |  | 1·073*** |
|  |  | (1·064-1·094) |  | (1·005-1·070) |  | (1·059-1·087) |
| Rate of hospital beds per 1,000 population (%) |  | 1·007 |  | 1·034** |  | 1·011* |
|  |  | (0·995-1·020) |  | (1·004-1·064) |  | (0·999-1·023) |
| Rate of physicians per 1,000 population (%) |  | 1·002 |  | 0·989 |  | 1·000 |
|  |  | (0·991-1·013) |  | (0·965-1·014) |  | (0·989-1·010) |
| Rate of nurses per 1,000 population (%) |  | 0·928*** |  | 0·908*** |  | 0·925*** |
|  |  | (0·921-0·936) |  | (0·892-0·925) |  | (0·918-0·932) |
| Households with proper garbage collection (%) |  | 0·942*** |  | 0·897*** |  | 0·935*** |
|  |  | (0·928-0·956) |  | (0·866-0·928) |  | (0·922-0·949) |
| Year (2008) | 1·054*** | 1·032*** | 1·074*** | 1·045*** | 1·057*** | 1·034*** |
|  | (1·042-1·065) | (1·021-1·044) | (1·047-1·100) | (1·019-1·071) | (1·046-1·067) | (1·024-1·045) |
| Year (2013) | 0·993 | 1·000 | 0·961*** | 0·969** | 0·988** | 0·995 |
|  | (0·982-1·004) | (0·989-1·011) | (0·936-0·987) | (0·944-0·995) | (0·978-0·999) | (0·985-1·006) |
| Year (2015) | 0·929*** | 0·947*** | 0·824*** | 0·844*** | 0·914*** | 0·932*** |
|  | (0·918-0·940) | (0·936-0·958) | (0·801-0·848) | (0·821-0·869) | (0·904-0·924) | (0·922-0·942) |

**Source:** Author’s data analysis for 87,962 observations – 5,507 municipalities in Brazil over 16 years (from 2004 to 2019).

**Note**: Data are in Rate Ratio (RR) coefficients (95% CI) unless otherwise specified; Time shocks are controls for specific years of economic crisis – 2008, 2013, and 2015; ***, ** and * denote significance at 1%, 5%, and 10% respectively. BFP=Bolsa Familia Program. PBC=Benefício de Prestação Continuada Program.

**Web Table 6– Empirical results for mortality models with filter for adequate information, 2000-2019, Brazil. (Incidence-Rate Ratios -- IRR)**

|  | **Under 1** | | **From 1 to 4** | | **Under 5** | |
| --- | --- | --- | --- | --- | --- | --- |
|  | **Without controls** | **With controls** | **Without controls** | **With controls** | **Without controls** | **With controls** |
| BFP target coverage |  |  |  |  |  |  |
| Low (0-30%) | 1 | 1 | 1 | 1 | 1 | 1 |
|  | (1·000-1·000) | (1·000-1·000) | (1·000-1·000) | (1·000-1·000) | (1·000-1·000) | (1·000-1·000) |
| Intermediate (30-70%) | 1·007 | 1·010 | 1·064* | 1·072* | 1·015 | 1·019 |
|  | (0·975-1·039) | (0·978-1·043) | (0·990-1·144) | (0·997-1·153) | (0·985-1·046) | (0·989-1·050) |
| High (70-100%) | 0·937*** | 0·954*** | 0·959 | 0·985 | 0·941*** | 0·959*** |
|  | (0·908-0·967) | (0·925-0·985) | (0·892-1·030) | (0·917-1·058) | (0·913-0·969) | (0·931-0·988) |
| Consolidated (100%) | 0·828*** | 0·876*** | 0·854*** | 0·928** | 0·832*** | 0·884*** |
|  | (0·803-0·853) | (0·850-0·903) | (0·799-0·914) | (0·866-0·994) | (0·809-0·856) | (0·859-0·909) |
| BPC municipal coverage |  |  |  |  |  |  |
| Low (0-33%) | 1 | 1 | 1 | 1 | 1 | 1 |
|  | (1·000-1·000) | (1·000-1·000) | (1·000-1·000) | (1·000-1·000) | (1·000-1·000) | (1·000-1·000) |
| Intermediate (33-66%) | 0·908*** | 0·916*** | 0·895*** | 0·906*** | 0·906*** | 0·914*** |
|  | (0·896-0·920) | (0·904-0·929) | (0·867-0·924) | (0·877-0·935) | (0·894-0·917) | (0·903-0·926) |
| Consolidated (66-100%) | 0·792*** | 0·837*** | 0·755*** | 0·813*** | 0·786*** | 0·833*** |
|  | (0·777-0·808) | (0·820-0·853) | (0·721-0·790) | (0·775-0·852) | (0·772-0·801) | (0·817-0·849) |
| Proportion of individuals older than 15 years who are illiterate (%) |  | 1·028** |  | 1·049* |  | 1·031*** |
|  |  | (1·005-1·052) |  | (0·996-1·104) |  | (1·010-1·053) |
| Poverty rate (%) |  | 1·054*** |  | 1·084*** |  | 1·057*** |
|  |  | (1·036-1·072) |  | (1·042-1·127) |  | (1·041-1·074) |
| Urbanization rate (%) |  | 0·956*** |  | 0·900*** |  | 0·948*** |
|  |  | (0·928-0·986) |  | (0·839-0·964) |  | (0·921-0·975) |
| Fertility rate (%) |  | 1·080*** |  | 1·046* |  | 1·075*** |
|  |  | (1·059-1·102) |  | (0·998-1·095) |  | (1·055-1·096) |
| Rate of hospital beds per 1,000 population (%) |  | 1·004 |  | 1·022 |  | 1·006 |
|  |  | (0·985-1·022) |  | (0·980-1·066) |  | (0·989-1·024) |
| Rate of physicians per 1,000 population (%) |  | 0·985 |  | 1·011 |  | 0·989 |
|  |  | (0·968-1·003) |  | (0·972-1·053) |  | (0·973-1·006) |
| Rate of nurses per 1,000 population (%) |  | 0·926*** |  | 0·901*** |  | 0·923*** |
|  |  | (0·915-0·938) |  | (0·875-0·928) |  | (0·912-0·934) |
| Households with proper garbage collection |  | 0·958*** |  | 0·914*** |  | 0·951*** |
|  |  | (0·937-0·980) |  | (0·868-0·962) |  | (0·932-0·971) |
| Year (2008) | 1·051*** | 1·035*** | 1·069*** | 1·047** | 1·053*** | 1·036*** |
|  | (1·034-1·068) | (1·018-1·052) | (1·030-1·109) | (1·009-1·087) | (1·038-1·069) | (1·021-1·052) |
| Year (2013) | 1·003 | 1·010 | 0·936*** | 0·947*** | 0·993 | 1·001 |
|  | (0·986-1·020) | (0·993-1·027) | (0·899-0·975) | (0·910-0·986) | (0·977-1·009) | (0·985-1·016) |
| Year (2015) | 0·939*** | 0·954*** | 0·818*** | 0·837*** | 0·921*** | 0·937*** |
|  | (0·923-0·955) | (0·938-0·970) | (0·784-0·854) | (0·802-0·874) | (0·907-0·936) | (0·922-0·952) |

**Source:** Author’s data analysis for 40,768 observations – 1,788 municipalities in Brazil over 20 years (from 2000 to 2019).

**Note**: Data are in Rate Ratio (RR) coefficients; Confidence intervals in parentheses; Time shocks are controls for specific years of economic crisis -- 2008, 2013, and 2015; ***, ** and * denote significance at 1%, 5%, and 10% respectively. BFP=Bolsa Familia Program. PBC=Benefício de Prestação Continuada Program.

**Web Table 7 – Mortality models with additional variables – Omitted variable bias test. 2004-2019, Brazil. (Incidence-Rate Ratios -- IRR)**

|  | **Model I** | **Model II** | **Model III** | **Model IV** |
| --- | --- | --- | --- | --- |
|  | **Overall** | **I + Education (degrees)** | **II + Living conditions** | **III + Inequality** |
| BFP target coverage |  |  |  |  |
| Low (0-30%) | 1 | 1 | 1 | 1 |
|  | (1·000-1·000) | (1·000-1·000) | (1·000-1·000) | (1·000-1·000) |
| Intermediate (30-70%) | 1·008 | 1·011 | 1·012 | 1·011 |
|  | (0·976-1·041) | (0·979-1·044) | (0·980-1·045) | (0·979-1·044) |
| High (70-100%) | 0·952*** | 0·958*** | 0·960** | 0·960** |
|  | (0·922-0·983) | (0·928-0·989) | (0·930-0·991) | (0·930-0·991) |
| Consolidated (≥100%) | 0·868*** | 0·876*** | 0·881*** | 0·884*** |
|  | (0·842-0·894) | (0·850-0·903) | (0·855-0·908) | (0·857-0·911) |
| BPC municipal coverage |  | 1 | 1 | 1 |
| Low (0-33%) | 1 | (1·000-1·000) | (1·000-1·000) | (1·000-1·000) |
|  | (1·000-1·000) | 0·921*** | 0·922*** | 0·922*** |
| Intermediate (33-66%) | 0·920*** | (0·908-0·933) | (0·910-0·935) | (0·910-0·935) |
|  | (0·908-0·933) | 0·843*** | 0·847*** | 0·848*** |
| Consolidated (66-100%) | 0·843*** | (0·826-0·861) | (0·830-0·864) | (0·831-0·866) |
|  | (0·826-0·861) | 1·046*** | 1·047*** | 1·047*** |
| Proportion of individuals older than 15 years who are illiterate (%) | 1·053*** | (1·024-1·068) | (1·025-1·069) | (1·025-1·069) |
|  | (1·032-1·075) | 1·034*** | 1·031*** | 1·032*** |
| Poverty rate (%) | 1·034*** | (1·018-1·050) | (1·015-1·047) | (1·016-1·048) |
|  | (1·018-1·050) | 0·941*** | 0·944*** | 0·945*** |
| Urbanization rate (%) | 0·934*** | (0·914-0·969) | (0·917-0·972) | (0·918-0·974) |
|  | (0·907-0·962) | 1·050*** | 1·050*** | 1·051*** |
| Fertility rate (%) | 1·057*** | (1·031-1·070) | (1·031-1·070) | (1·032-1·071) |
|  | (1·038-1·076) | 0·999 | 1·000 | 0·999 |
| Rate of hospital beds per 1,000 population (%) | 1·002 | (0·981-1·018) | (0·981-1·018) | (0·981-1·018) |
|  | (0·983-1·020) | 0·986 | 0·986 | 0·987 |
| Rate of physicians per 1,000 population (%) | 0·984* | (0·969-1·003) | (0·969-1·004) | (0·970-1·005) |
|  | (0·966-1·001) | 0·937*** | 0·938*** | 0·939*** |
| Rate of nurses per 1,000 population (%) | 0·933*** | (0·925-0·949) | (0·926-0·950) | (0·927-0·951) |
|  | (0·921-0·945) | 0·977** | 0·981* | 0·982 |
| Households with proper garbage collection (%) | 0·966*** | (0·956-0·998) | (0·960-1·003) | (0·961-1·004) |
|  | (0·945-0·987) | 1·035*** | 1·030*** | 1·029*** |
| Year (2008) | 1·032*** | (1·018-1·052) | (1·013-1·047) | (1·012-1·046) |
|  | (1·015-1·049) | 1·006 | 1·008 | 1·008 |
| Year (2013) | 1·008 | (0·989-1·023) | (0·991-1·025) | (0·991-1·025) |
|  | (0·992-1·025) | 0·952*** | 0·954*** | 0·955*** |
| Year (2015) | 0·953*** | (0·936-0·968) | (0·938-0·971) | (0·938-0·971) |
|  | (0·936-0·969) | 0·970*** | 0·972*** | 0·974*** |
| Primary education (%) |  | (0·953-0·986) | (0·955-0·989) | (0·957-0·990) |
|  |  | 0·962*** | 0·963*** | 0·965*** |
| High school education (%) |  | (0·944-0·979) | (0·946-0·981) | (0·947-0·982) |
|  |  |  | 0·972*** | 0·972*** |
| Households with inadequate sanitation (%) |  |  | (0·953-0·991) | (0·953-0·991) |
|  |  |  | 1·012** | 1·012** |
| Households with piped water (%) |  |  | (1·002-1·023) | (1·002-1·023) |
|  |  |  |  | 1·059*** |
| Gini Index (%) |  |  |  | (1·028-1·091) |
|  | 0·142*** | 0·146*** | 0·146*** | 0·145*** |
|  | (0·124-0·163) | (0·128-0·168) | (0·127-0·168) | (0·126-0·167) |

**Source:** Author’s data analysis for 40,768 observations – 2,548 municipalities in Brazil over 16 years (from 2004 to 2019).

**Note**: Data are in Rate Ratio (RR) coefficients (95% CI) unless otherwise specified; Time shocks are controls for specific years of economic crisis – 2008, 2013, and 2015; ***, ** and * denote significance at 1%, 5%, and 10% respectively. Educations variables are the percentage of population with primary and high school attainment; Sewage is the percentage of households with adequate sewage collection; Water is the percentage of households with access to treated water; GINI is the income inequality index, and; Nurse rate is the rate of nurses per 1,000 population. BFP=Bolsa Familia Program. PBC=Benefício de Prestação Continuada Program.

**Web Table 8 - Empirical results for mortality models using continuous variables (with filter for adequate information), 2004-2019, Brazil.**

|  | **Under 1** | | **From 1 to 4** | | **Under 5** | | |  |
| --- | --- | --- | --- | --- | --- | --- | --- | --- |
|  | **Crude** | **Adjusted** | **Crude** | **Adjusted** | | **Crude** | **Adjusted** | |
| BFP target coverage (%) | 0·998*** | 0·999*** | 0·998*** | 0·999** | | 0·998*** | 0·999*** | |
|  | (0·998-0·998) | (0·999-0·999) | (0·998-0·999) | (0·999-1·000) | | (0·998-0·998) | (0·999-0·999) | |
| BPC municipal coverage (%) | 0·856*** | 0·976*** | 0·821*** | 0·953*** | | 0·850*** | 0·972*** | |
|  | (0·849-0·864) | (0·964-0·989) | (0·804-0·839) | (0·927-0·981) | | (0·843-0·858) | (0·961-0·984) | |
| Proportion of individuals older than 15 years who are illiterate (%) |  | 1·006*** |  | 1·010*** | |  | 1·007*** | |
|  |  | (1·003-1·010) |  | (1·003-1·017) | |  | (1·004-1·010) | |
| Poverty rate (%) |  | 1·005*** |  | 1·007*** | |  | 1·005*** | |
|  |  | (1·004-1·006) |  | (1·004-1·010) | |  | (1·004-1·007) | |
| Urbanization rate (%) |  | 0·997*** |  | 0·998 | |  | 0·997*** | |
|  |  | (0·996-0·999) |  | (0·994-1·001) | |  | (0·996-0·999) | |
| Fertility rate (%) |  | 1·222*** |  | 1·142*** | |  | 1·209*** | |
|  |  | (1·173-1·273) |  | (1·042-1·251) | |  | (1·165-1·256) | |
| Rate of hospital beds per 1,000 population (%) |  | 1-003 |  | 0·999 | |  | 1-003 | |
|  |  | (0·997-1·009) |  | (0·983-1·015) | |  | (0·997-1·008) | |
| Rate of physicians per 1,000 population (%) |  | 0·966*** |  | 0·960** | |  | 0·966*** | |
|  |  | (0·951-0·982) |  | (0·925-0·996) | |  | (0·952-0·980) | |
| Rate of nurses per 1,000 population (%) |  | 0·982* |  | 0·943** | |  | 0·976** | |
|  |  | (0·962-1·003) |  | (0·899-0·988) | |  | (0·958-0·995) | |
| Households with proper garbage collection (%) |  | 1·002*** |  | 1-001 | |  | 1·002*** | |
|  |  | (1·001-1·003) |  | (0·999-1·004) | |  | (1·001-1·003) | |
| Year (2008) | 1·019** | 0·990 | 1·029 | 0·998 | | 1·020** | 0·991 | |
|  | (1·003-1·035) | (0·974-1·007) | (0·991-1·068) | (0·960-1·036) | | (1·005-1·035) | (0·976-1·006) | |
| Year (2013) | 0·998 | 1·006 | 0·936*** | 0·944*** | | 0·989 | 0·997 | |
|  | (0·982-1·015) | (0·990-1·023) | (0·899-0·974) | (0·906-0·983) | | (0·974-1·005) | (0·982-1·013) | |
| Year (2015) | 0·948*** | 0·975*** | 0·832*** | 0·860*** | | 0·931*** | 0·958*** | |
|  | (0·931-0·964) | (0·958-0·992) | (0·798-0·868) | (0·823-0·898) | | (0·916-0·946) | (0·943-0·974) | |
| Intercept | 0·172*** | 0·0787*** | 0·106*** | 0·0487*** | | 0·180*** | 0·0866*** | |
|  | (0·152-0·194) | (0·062-0·101) | (0·074-0·150) | (0·027-0·089) | | (0·161-0·201) | (0·069-0·108) | |
| **Number of observations** | 40,730 | 40,730 | 38,634 | 38,634 | | 40,762 | 40,762 | |

**Source:** Author’s data analysis for 40,768 observations – 2,548 municipalities in Brazil over 16 years (from 2004 to 2019).

**Note**: Data are in Rate Ratio (RR) coefficients (95% CI) unless otherwise specified; Time shocks are controls for specific years of economic crisis – 2008, 2013, and 2015; ***, ** and * denote significance at 1%, 5%, and 10% respectively. BFP=Bolsa Familia Program. PBC=Benefício de Prestação Continuada Program.

**Web Table 9 –** **Colinearity test, without poverty, 2004-2019, Brazil.**

|  | **Under 1** | | **From 1 to 4** | | **Under 5** | |
| --- | --- | --- | --- | --- | --- | --- |
|  | **Crude** | **Adjusted** | **Crude** | **Adjusted** | **Crude** | **Adjusted** |
| BFP target coverage |  |  |  |  |  |  |
| Low (0-30%) | 1 | 1 | 1 | 1 | 1 | 1 |
|  | (1·000-1·000) | (1·000-1·000) | (1·000-1·000) | (1·000-1·000) | (1·000-1·000) | (1·000-1·000) |
| Intermediate (30-70%) | 1-008 | 1-009 | 1·068* | 1·070* | 1-017 | 1-017 |
|  | (0·976-1·041) | (0·977-1·041) | (0·993-1·148) | (0·995-1·150) | (0·987-1·047) | (0·988-1·048) |
| High (70-100%) | 0·952*** | 0·952*** | 0·980 | 0·980 | 0·956*** | 0·956*** |
|  | (0·922-0·983) | (0·922-0·983) | (0·912-1·053) | (0·912-1·053) | (0·928-0·985) | (0·928-0·985) |
| Consolidated (≥100) | 0·868*** | 0·865*** | 0·914*** | 0·910*** | 0·874*** | 0·872*** |
|  | (0·842-0·894) | (0·840-0·892) | (0·854-0·979) | (0·850-0·975) | (0·850-0·899) | (0·848-0·897) |
| BPC municipal coverage |  |  |  |  |  |  |
| Low (0-33%) | 1 | 1 | 1 | 1 | 1 | 1 |
|  | (1·000-1·000) | (1·000-1·000) | (1·000-1·000) | (1·000-1·000) | (1·000-1·000) | (1·000-1·000) |
| Intermediate (33-66%) | 0·920*** | 0·920*** | 0·913*** | 0·913*** | 0·919*** | 0·919*** |
|  | (0·908-0·933) | (0·908-0·933) | (0·884-0·943) | (0·884-0·943) | (0·907-0·931) | (0·907-0·931) |
| Consolidated (66-100%) | 0·843*** | 0·839*** | 0·822*** | 0·814*** | 0·840*** | 0·836*** |
|  | (0·826-0·861) | (0·823-0·857) | (0·783-0·862) | (0·776-0·854) | (0·824-0·856) | (0·820-0·852) |
| Proportion of individuals older than 15 years who are illiterate (%) | 1·053*** | 1·060*** | 1·062** | 1·075*** | 1·054*** | 1·062*** |
|  | (1·032-1·075) | (1·038-1·082) | (1·013-1·114) | (1·025-1·127) | (1·034-1·075) | (1·042-1·082) |
| Poverty rate (%) | 1·034*** |  | 1·067*** |  | 1·038*** |  |
|  | (1·018-1·050) |  | (1·029-1·107) |  | (1·023-1·053) |  |
| Urbanization rate (%) | 0·934*** | 0·934*** | 0·925** | 0·925** | 0·933*** | 0·933*** |
|  | (0·907-0·962) | (0·907-0·962) | (0·865-0·990) | (0·865-0·990) | (0·908-0·959) | (0·908-0·959) |
| Fertility rate (%) | 1·057*** | 1·065*** | 1·039* | 1·055** | 1·054*** | 1·064*** |
|  | (1·038-1·076) | (1·046-1·084) | (0·996-1·084) | (1·013-1·100) | (1·036-1·072) | (1·046-1·082) |
| Rate of hospital beds per 1,000 population (%) | 1-002 | 1-005 | 1-001 | 1-008 | 1-001 | 1-005 |
|  | (0·983-1·020) | (0·987-1·023) | (0·959-1·044) | (0·966-1·051) | (0·985-1·019) | (0·988-1·022) |
| Rate of physicians per 1,000 population (%) | 0·984* | 0·984* | 1-009 | 1-009 | 0·987 | 0·987 |
|  | (0·966-1·001) | (0·967-1·001) | (0·969-1·050) | (0·970-1·050) | (0·971-1·004) | (0·971-1·004) |
| Rate of nurses per 1,000 population (%) | 0·933*** | 0·931*** | 0·898*** | 0·894*** | 0·928*** | 0·925*** |
|  | (0·921-0·945) | (0·919-0·943) | (0·872-0·925) | (0·869-0·921) | (0·917-0·939) | (0·914-0·936) |
| Households with proper garbage collection (%) | 0·966*** | 0·965*** | 0·929*** | 0·927*** | 0·960*** | 0·958*** |
|  | (0·945-0·987) | (0·944-0·985) | (0·885-0·976) | (0·883-0·973) | (0·941-0·979) | (0·940-0·978) |
| Year (2008) | 1·032*** | 1·030*** | 1·044** | 1·040** | 1·033*** | 1·031*** |
|  | (1·015-1·049) | (1·013-1·046) | (1·006-1·084) | (1·002-1·079) | (1·018-1·049) | (1·016-1·047) |
| Year (2013) | 1-008 | 1-005 | 0·947*** | 0·941*** | 0·999 | 0·995 |
|  | (0·992-1·025) | (0·988-1·022) | (0·909-0·986) | (0·904-0·980) | (0·984-1·015) | (0·980-1·011) |
| Year (2015) | 0·953*** | 0·949*** | 0·838*** | 0·833*** | 0·936*** | 0·933*** |
|  | (0·936-0·969) | (0·933-0·966) | (0·803-0·875) | (0·798-0·869) | (0·921-0·951) | (0·918-0·948) |
| Intercept | 0·142*** | 0·143*** | 0·0717*** | 0·0729*** | 0·146*** | 0·147*** |
|  | (0·124-0·163) | (0·125-0·164) | (0·050-0·103) | (0·051-0·104) | (0·130-0·165) | (0·131-0·166) |
| **Number of observations** | **40,730** | **40,730** | **38,634** | **38,634** | **40,762** | **40,762** |

**Source:** Author’s data analysis for 40,768 observations – 2,548 municipalities in Brazil over 16 years (from 2004 to 2019).

**Note**: Data are in Rate Ratio (RR) coefficients (95% CI) unless otherwise specified; Time shocks are controls for specific years of economic crisis – 2008, 2013, and 2015; ***, ** and * denote significance at 1%, 5%, and 10% respectively. BFP=Bolsa Familia Program. PBC=Benefício de Prestação Continuada Program.

**Web Table 10 – Empirical results for mortality models with different time shocks, 2004-2019, Brazil.**

|  | **No time control** | **Model I (below 1)** | **Model II** | **Model III** | **Model IV** | **Model V** |
| --- | --- | --- | --- | --- | --- | --- |
| BFP target coverage |  |  |  |  |  |  |
| Low (0-30%) | 1 | 1 | 1 | 1 | 1 | 1 |
|  | (1·000-1·000) | (1·000-1·000) | (1·000-1·000) | (1·000-1·000) | (1·000-1·000) | (1·000-1·000) |
| Intermediate (30-70%) | 1·008 | 1·008 | 1·007 | 1·008 | 1·004 | 1·009 |
|  | (0·976-1·041) | (0·976-1·041) | (0·975-1·040) | (0·977-1·041) | (0·973-1·037) | (0·977-1·042) |
| High (70-100%) | 0·955*** | 0·952*** | 0·948*** | 0·954*** | 0·936*** | 0·954*** |
|  | (0·925-0·986) | (0·922-0·983) | (0·918-0·979) | (0·924-0·985) | (0·906-0·966) | (0·924-0·985) |
| Consolidated (≥100%) | 0·873*** | 0·868*** | 0·861*** | 0·873*** | 0·856*** | 0·871*** |
|  | (0·847-0·899) | (0·842-0·894) | (0·835-0·887) | (0·847-0·900) | (0·831-0·883) | (0·845-0·898) |
| BPC municipal coverage |  |  |  |  |  |  |
| Low (0-33%) | 1 | 1 | 1 | 1 | 1 | 1 |
|  | (1·000-1·000) | (1·000-1·000) | (1·000-1·000) | (1·000-1·000) | (1·000-1·000) | (1·000-1·000) |
| Intermediate (33-66%) | 0·917*** | 0·920*** | 0·922*** | 0·919*** | 0·927*** | 0·922*** |
|  | (0·904-0·929) | (0·908-0·933) | (0·909-0·935) | (0·907-0·932) | (0·915-0·940) | (0·909-0·934) |
| Consolidated (66-100%) | 0·836*** | 0·843*** | 0·845*** | 0·840*** | 0·851*** | 0·845*** |
|  | (0·819-0·853) | (0·826-0·861) | (0·828-0·862) | (0·823-0·857) | (0·834-0·869) | (0·828-0·863) |
| Proportion of individuals older than 15 years who are illiterate (%) | 1·053*** | 1·053*** | 1·052*** | 1·051*** | 1·052*** | 1·048*** |
|  | (1·032-1·076) | (1·032-1·075) | (1·031-1·074) | (1·030-1·073) | (1·030-1·074) | (1·026-1·070) |
| Poverty rate (%) | 1·035*** | 1·034*** | 1·029*** | 1·033*** | 1·029*** | 1·037*** |
|  | (1·019-1·051) | (1·018-1·050) | (1·013-1·045) | (1·018-1·049) | (1·013-1·045) | (1·021-1·053) |
| Urbanization rate (%) | 0·933*** | 0·934*** | 0·937*** | 0·934*** | 0·935*** | 0·934*** |
|  | (0·906-0·961) | (0·907-0·962) | (0·910-0·964) | (0·907-0·962) | (0·908-0·963) | (0·907-0·962) |
| Fertility rate (%) | 1·060*** | 1·057*** | 1·056*** | 1·059*** | 1·059*** | 1·056*** |
|  | (1·041-1·079) | (1·038-1·076) | (1·037-1·076) | (1·040-1·078) | (1·040-1·078) | (1·037-1·076) |
| Rate of hospital beds per 1,000 population (%) | 1·003 | 1·002 | 1·001 | 1·002 | 1·002 | 1·000 |
|  | (0·985-1·021) | (0·983-1·020) | (0·983-1·020) | (0·984-1·021) | (0·984-1·021) | (0·982-1·018) |
| Rate of physicians per 1,000 population (%) | 0·983* | 0·984* | 0·983* | 0·983* | 0·983* | 0·985* |
|  | (0·965-1·000) | (0·966-1·001) | (0·965-1·000) | (0·966-1·000) | (0·966-1·000) | (0·968-1·003) |
| Rate of nurses per 1,000 population (%) | 0·929*** | 0·933*** | 0·934*** | 0·931*** | 0·937*** | 0·935*** |
|  | (0·917-0·941) | (0·921-0·945) | (0·922-0·946) | (0·919-0·943) | (0·925-0·949) | (0·923-0·947) |
| Households with proper garbage collection (%) | 0·965*** | 0·966*** | 0·967*** | 0·966*** | 0·968*** | 0·969*** |
|  | (0·944-0·986) | (0·945-0·987) | (0·946-0·987) | (0·946-0·987) | (0·948-0·989) | (0·948-0·990) |
| Year (2008) |  | 1·032*** |  |  | 1·041*** |  |
|  |  | (1·015-1·049) |  |  | (1·024-1·058) |  |
| Year (2013) |  | 1·008 |  |  |  |  |
|  |  | (0·992-1·025) |  |  |  |  |
| Year (2015) |  | 0·953*** |  |  |  |  |
|  |  | (0·936-0·969) |  |  |  |  |
| Year (2007) |  |  | 1·067*** |  |  |  |
|  |  |  | (1·050-1·084) |  |  |  |
| Year (2011) |  |  | 0·990 | 0·983** | 0·992 |  |
|  |  |  | (0·973-1·006) | (0·966-0·999) | (0·975-1·009) |  |
| Year (2016) |  |  | 0·991 |  |  | 0·981** |
|  |  |  | (0·974-1·008) |  |  | (0·964-0·998) |
| Year (2014) |  |  |  | 1·005 |  | 1·002 |
|  |  |  |  | (0·988-1·022) |  | (0·986-1·019) |
| Year (2018) |  |  |  | 0·955*** |  |  |
|  |  |  |  | (0·938-0·972) |  |  |
| Year (2006) |  |  |  |  | 1·062*** |  |
|  |  |  |  |  | (1·045-1·079) |  |
| Year (2018) |  |  |  |  |  | 0·922*** |
|  |  |  |  |  |  | (0·905-0·938) |
| Intercept | 0·140*** | 0·142*** | 0·143*** | 0·142*** | 0·142*** | 0·143*** |
|  | (0·123-0·160) | (0·124-0·163) | (0·125-0·164) | (0·124-0·162) | (0·124-0·163) | (0·125-0·164) |

**Source:** Author’s data analysis for 40,768 observations – 2,548 municipalities in Brazil over 16 years (from 2004 to 2019).

**Note**: Data are in Rate Ratio (RR) coefficients (95% CI) unless otherwise specified; Time shocks are controls for specific years – 2006, 2008, 2011, 2014, 2013, 2014, 2015, 2016, and 2018; ***, ** and * denote significance at 1%, 5%, and 10% respectively. BFP=Bolsa Familia Program. PBC=Benefício de Prestação Continuada Program.

**Web Table 11 – Empirical results for mortality models estimated using Negative Binomial and Poisson regression, 2004-2019, Brazil.**

|  | **Under 1** | | **From 1 to 4** | | **Under 5** | |
| --- | --- | --- | --- | --- | --- | --- |
|  | **Negative Binomial** | **Poisson** | **Negative Binomial** | **Poisson** | **Negative Binomial** | **Poisson** |
| BFP target coverage |  |  |  |  |  |  |
| Low (0-30%) | 1 | 1 | 1 | 1 | 1 | 1 |
|  | (1·000-1·000) | (1·000-1·000) | (1·000-1·000) | (1·000-1·000) | (1·000-1·000) | (1·000-1·000) |
| Intermediate (30-70%) | 1·008 | 1·009 | 1·068* | 1·068 | 1·017 | 1·018 |
|  | (0·976-1·041) | (0·967-1·053) | (0·993-1·148) | (0·985-1·159) | (0·987-1·047) | (0·976-1·062) |
| High (70-100%) | 0·952*** | 0·953** | 0·980 | 0·979 | 0·956*** | 0·957** |
|  | (0·922-0·983) | (0·917-0·990) | (0·912-1·053) | (0·905-1·059) | (0·928-0·985) | (0·922-0·993) |
| Consolidated (≥100%) | 0·868*** | 0·868*** | 0·914*** | 0·915** | 0·874*** | 0·874*** |
|  | (0·842-0·894) | (0·837-0·899) | (0·854-0·979) | (0·845-0·990) | (0·850-0·899) | (0·843-0·907) |
| BPC municipal coverage |  |  |  |  |  |  |
| Low (0-33%) | 1 | 1 | 1 | 1 | 1 | 1 |
|  | (1·000-1·000) | (1·000-1·000) | (1·000-1·000) | (1·000-1·000) | (1·000-1·000) | (1·000-1·000) |
| Intermediate (33-66%) | 0·920*** | 0·921*** | 0·913*** | 0·913*** | 0·919*** | 0·920*** |
|  | (0·908-0·933) | (0·892-0·950) | (0·884-0·943) | (0·854-0·975) | (0·907-0·931) | (0·889-0·952) |
| Consolidated (66-100%) | 0·843*** | 0·844*** | 0·822*** | 0·821*** | 0·840*** | 0·841*** |
|  | (0·826-0·861) | (0·806-0·883) | (0·783-0·862) | (0·759-0·888) | (0·824-0·856) | (0·802-0·881) |
| Proportion of individuals older than 15 years who are illiterate (%) | 1·053*** | 1·054*** | 1·062** | 1·063** | 1·054*** | 1·055*** |
|  | (1·032-1·075) | (1·017-1·092) | (1·013-1·114) | (1·008-1·122) | (1·034-1·075) | (1·020-1·092) |
| Poverty rate (%) | 1·034*** | 1·035*** | 1·067*** | 1·068*** | 1·038*** | 1·039*** |
|  | (1·018-1·050) | (1·009-1·061) | (1·029-1·107) | (1·025-1·114) | (1·023-1·053) | (1·015-1·065) |
| Urbanization rate (%) | 0·934*** | 0·935*** | 0·925** | 0·926** | 0·933*** | 0·933*** |
|  | (0·907-0·962) | (0·894-0·978) | (0·865-0·990) | (0·866-0·990) | (0·908-0·959) | (0·895-0·973) |
| Fertility rate (%) | 1·057*** | 1·056*** | 1·039* | 1·039 | 1·054*** | 1·053*** |
|  | (1·038-1·076) | (1·023-1·089) | (0·996-1·084) | (0·986-1·095) | (1·036-1·072) | (1·021-1·086) |
| Rate of hospital beds per 1,000 population (%) | 1·002 | 1·003 | 1·001 | 0·999 | 1·001 | 1·002 |
|  | (0·983-1·020) | (0·972-1·034) | (0·959-1·044) | (0·956-1·043) | (0·985-1·019) | (0·973-1·032) |
| Rate of physicians per 1,000 population (%) | 0·984* | 0·985 | 1·009 | 1·007 | 0·987 | 0·988 |
|  | (0·966-1·001) | (0·967-1·003) | (0·969-1·050) | (0·968-1·047) | (0·971-1·004) | (0·971-1·006) |
| Rate of nurses per 1,000 population (%) | 0·933*** | 0·932*** | 0·898*** | 0·899*** | 0·928*** | 0·927*** |
|  | (0·921-0·945) | (0·913-0·951) | (0·872-0·925) | (0·871-0·927) | (0·917-0·939) | (0·910-0·945) |
| Households with proper garbage collection (%) | 0·966*** | 0·965* | 0·929*** | 0·927*** | 0·960*** | 0·959** |
|  | (0·945-0·987) | (0·931-1·001) | (0·885-0·976) | (0·881-0·976) | (0·941-0·979) | (0·928-0·992) |
| Year (2008) | 1·032*** | 1·032*** | 1·044** | 1·044** | 1·033*** | 1·034*** |
|  | (1·015-1·049) | (1·017-1·047) | (1·006-1·084) | (1·006-1·082) | (1·018-1·049) | (1·019-1·049) |
| Year (2013) | 1·008 | 1·009 | 0·947*** | 0·947*** | 0·999 | 1·000 |
|  | (0·992-1·025) | (0·991-1·027) | (0·909-0·986) | (0·912-0·983) | (0·984-1·015) | (0·984-1·017) |
| Year (2015) | 0·953*** | 0·953*** | 0·838*** | 0·838*** | 0·936*** | 0·937*** |
|  | (0·936-0·969) | (0·938-0·968) | (0·803-0·875) | (0·804-0·874) | (0·921-0·951) | (0·923-0·950) |

**Source:** Author’s data analysis for 40,768 observations – 2,548 municipalities in Brazil over 16 years (from 2004 to 2019).

**Note**: Data are in Rate Ratio (RR) coefficients (95% CI) unless otherwise specified; Time shocks are controls for specific years of economic crisis – 2008, 2013, and 2015; ***, ** and * denote significance at 1%, 5%, and 10% respectively. BFP=Bolsa Familia Program. PBC=Benefício de Prestação Continuada Program.

**Web Table 12 – Empirical results for poverty levels by quartiles on Children mortality models, 2004-2019, Brazil.**

|  | **Under 1** | | | | **1 - 4** | | | | **Under-5** | | | |
| --- | --- | --- | --- | --- | --- | --- | --- | --- | --- | --- | --- | --- |
|  | **Poverty quartiles** | | | | | | | | | | | |
|  | **Q1** | **Q2** | **Q3** | **Q4** | **Q1** | **Q2** | **Q3** | **Q4** | **Q1** | **Q2** | **Q3** | **Q4** |
|  | **Below 2.16** | **2.16 - 8.63** | **8.63 - 23.34** | **Above 23.34** | **Below 2.16** | **2.16 - 8.63** | **8.63 - 23.34** | **Above 23.34** | **Below 2.16** | **2.16 - 8.63** | **8.63 - 23.34** | **Above 23.34** |
| BFP target coverage |  |  |  |  |  |  |  |  |  |  |  |  |
| Low (0-30%) | 1 | 1 | 1 | 1 | 1 | 1 | 1 | 1 | 1 | 1 | 1 | 1 |
|  | (1·000-1·000) | (1·000-1·000) | (1·000-1·000) | (1·000-1·000) | (1·000-1·000) | (1·000-1·000) | (1·000-1·000) | (1·000-1·000) | (1·000-1·000) | (1·000-1·000) | (1·000-1·000) | (1·000-1·000) |
| Intermediate (30-70%) | 2-759 | 0·958 | 0·993 | 0·999 | 1351057·1 | 1-042 | 1-058 | 1-069 | 3·479* | 0·969 | 1-001 | 1-013 |
|  | (0·635-11·995) | (0·900-1·021) | (0·936-1·053) | (0·944-1·059) | (0·000-·) | (0·895-1·213) | (0·921-1·215) | (0·949-1·205) | (0·809-14·960) | (0·914-1·028) | (0·948-1·058) | (0·960-1·068) |
| High (70-100%) | 2-274 | 0·954 | 0·962 | 0·918*** | 1026788·3 | 1-038 | 0·963 | 0·959 | 2-847 | 0·965 | 0·962 | 0·927*** |
|  | (0·547-9·460) | (0·901-1·010) | (0·906-1·021) | (0·867-0·973) | (0·000-·) | (0·903-1·193) | (0·836-1·110) | (0·851-1·081) | (0·690-11·748) | (0·915-1·018) | (0·910-1·017) | (0·878-0·977) |
| Consolidated (≥100%) | 2-185 | 0·914*** | 0·874*** | 0·812*** | 1464118·2 | 1-022 | 0·997 | 0·838*** | 2-851 | 0·928*** | 0·891*** | 0·818*** |
|  | (0·530-9·016) | (0·866-0·964) | (0·826-0·926) | (0·767-0·860) | (0·000-·) | (0·896-1·166) | (0·872-1·140) | (0·744-0·944) | (0·696-11·674) | (0·882-0·975) | (0·845-0·940) | (0·776-0·863) |
| BPC municipal coverage |  |  |  |  |  |  |  |  |  |  |  |  |
| Low (0-33%) | 1 | 1 | 1 | 1 | 1 | 1 | 1 | 1 | 1 | 1 | 1 | 1 |
|  | (1·000-1·000) | (1·000-1·000) | (1·000-1·000) | (1·000-1·000) | (1·000-1·000) | (1·000-1·000) | (1·000-1·000) | (1·000-1·000) | (1·000-1·000) | (1·000-1·000) | (1·000-1·000) | (1·000-1·000) |
| Intermediate (33-66%) | 0·917*** | 0·886*** | 1·000 | 0·914*** | 0·833*** | 0·852*** | 0·965 | 1-008 | 0·905*** | 0·881*** | 0·994 | 0·927*** |
|  | (0·877-0·958) | (0·868-0·904) | (0·959-1·043) | (0·882-0·947) | (0·747-0·930) | (0·811-0·895) | (0·873-1·066) | (0·932-1·090) | (0·869-0·944) | (0·865-0·898) | (0·956-1·034) | (0·897-0·958) |
| Consolidated (66-100%) | 0·861*** | 0·812*** | 0·916*** | 0·811*** | 0·739*** | 0·720*** | 0·849** | 0·910* | 0·844*** | 0·799*** | 0·906*** | 0·826*** |
|  | (0·805-0·922) | (0·782-0·842) | (0·869-0·965) | (0·774-0·851) | (0·623-0·877) | (0·659-0·787) | (0·750-0·962) | (0·819-1·011) | (0·792-0·900) | (0·772-0·826) | (0·863-0·951) | (0·790-0·863) |
| Proportion of individuals older than 15 years who are illiterate (%) | 1·047 | 1·068** | 1·081*** | 1·078* | 0·952 | 1·043 | 1·156*** | 1·080 | 1·032 | 1·064** | 1·092*** | 1·079** |
|  | (0·954-1·150) | (1·011-1·128) | (1·047-1·116) | (0·998-1·164) | (0·759-1·194) | (0·916-1·188) | (1·071-1·247) | (0·924-1·263) | (0·946-1·126) | (1·012-1·119) | (1·060-1·125) | (1·005-1·158) |
| Poverty rate (%) | 1 | 1 | 1 | 1 | 1 | 1 | 1 | 1 | 1 | 1 | 1 | 1 |
|  | (1·000-1·000) | (1·000-1·000) | (1·000-1·000) | (1·000-1·000) | (1·000-1·000) | (1·000-1·000) | (1·000-1·000) | (1·000-1·000) | (1·000-1·000) | (1·000-1·000) | (1·000-1·000) | (1·000-1·000) |
| Urbanization rate (%) | 0·980 | 0·911* | 0·967 | 0·942** | 0·905 | 0·884 | 1·001 | 0·891* | 0·971 | 0·905** | 0·971 | 0·933*** |
|  | (0·842-1·141) | (0·827-1·003) | (0·909-1·029) | (0·892-0·993) | (0·624-1·312) | (0·703-1·112) | (0·866-1·155) | (0·790-1·004) | (0·842-1·119) | (0·827-0·989) | (0·917-1·028) | (0·888-0·981) |
| Fertility rate (%) | 1·086 | 1·059** | 1·069*** | 1·094*** | 1·264* | 1·014 | 1·024 | 1·257*** | 1·115** | 1·053** | 1·063*** | 1·117*** |
|  | (0·974-1·211) | (1·008-1·113) | (1·039-1·100) | (1·029-1·164) | (0·990-1·613) | (0·902-1·141) | (0·957-1·095) | (1·090-1·450) | (1·008-1·232) | (1·006-1·103) | (1·035-1·092) | (1·054-1·183) |
| Rate of hospital beds per 1,000 population (%) | 1·002 | 0·997 | 1·041 | 1·004 | 0·973 | 0·973 | 1·085 | 1·016 | 0·998 | 0·994 | 1·046** | 1·005 |
|  | (0·952-1·055) | (0·963-1·033) | (0·991-1·092) | (0·961-1·050) | (0·858-1·104) | (0·895-1·059) | (0·970-1·214) | (0·922-1·119) | (0·952-1·047) | (0·962-1·027) | (1·000-1·094) | (0·964-1·047) |
| Rate of physicians per 1,000 population (%) | 0·990 | 1·013 | 1·004 | 0·969** | 0·858** | 1·151** | 1·013 | 0·998 | 0·970 | 1·031 | 1·005 | 0·974* |
|  | (0·939-1·043) | (0·969-1·059) | (0·967-1·042) | (0·940-1·000) | (0·755-0·975) | (1·034-1·281) | (0·930-1·104) | (0·934-1·067) | (0·923-1·019) | (0·989-1·074) | (0·971-1·040) | (0·947-1·002) |
| Rate of nurses per 1,000 population (%) | 0·960** | 0·945*** | 0·945*** | 0·937*** | 0·954 | 0·904*** | 0·916*** | 0·887*** | 0·959** | 0·939*** | 0·941*** | 0·929*** |
|  | (0·928-0·993) | (0·920-0·971) | (0·920-0·971) | (0·911-0·963) | (0·877-1·037) | (0·848-0·964) | (0·860-0·976) | (0·835-0·942) | (0·929-0·990) | (0·916-0·963) | (0·918-0·965) | (0·906-0·953) |
| Households with proper garbage collection (%) | 0·962 | 0·950 | 0·977 | 0·963 | 1·041 | 1·070 | 0·933 | 0·905* | 0·973 | 0·964 | 0·970 | 0·954** |
|  | (0·869-1·066) | (0·886-1·019) | (0·935-1·021) | (0·920-1·008) | (0·821-1·321) | (0·902-1·270) | (0·843-1·034) | (0·818-1·001) | (0·885-1·069) | (0·903-1·029) | (0·931-1·010) | (0·914-0·995) |
| Year (2008) | 1·070*** | 1·049*** | 1·023 | 0·983 | 1·113** | 1·064 | 1·013 | 1·034 | 1·075*** | 1·051*** | 1·021 | 0·991 |
|  | (1·035-1·106) | (1·015-1·084) | (0·988-1·060) | (0·947-1·020) | (1·026-1·207) | (0·983-1·151) | (0·934-1·099) | (0·954-1·120) | (1·043-1·109) | (1·019-1·083) | (0·988-1·055) | (0·957-1·026) |
| Year (2013) | 1·005 | 1·037** | 1·032 | 1·029 | 0·981 | 0·934* | 0·986 | 0·980 | 1·002 | 1·023 | 1·025 | 1·020 |
|  | (0·978-1·034) | (1·008-1·068) | (0·985-1·082) | (0·982-1·078) | (0·915-1·053) | (0·869-1·004) | (0·882-1·102) | (0·882-1·088) | (0·976-1·029) | (0·996-1·051) | (0·982-1·071) | (0·976-1·065) |
| Year (2015) | 0·987 | 0·968** | 0·951* | 0·882*** | 0·861*** | 0·808*** | 0·901* | 0·943 | 0·969** | 0·946*** | 0·944** | 0·891*** |
|  | (0·957-1·019) | (0·943-0·993) | (0·904-1·001) | (0·835-0·932) | (0·793-0·934) | (0·755-0·864) | (0·799-1·016) | (0·839-1·061) | (0·941-0·999) | (0·923-0·969) | (0·900-0·989) | (0·847-0·938) |
| Intercept | 0·114*** | 0·250*** | 0·135*** | 0·131*** | 6·81e-08 | 0·104*** | 0·0627*** | 0·0480*** | 0·0799*** | 0·258*** | 0·151*** | 0·122*** |
|  | (0·024-0·530) | (0·153-0·408) | (0·100-0·183) | (0·103-0·168) | (0·000-·) | (0·029-0·375) | (0·028-0·141) | (0·028-0·082) | (0·018-0·353) | (0·166-0·402) | (0·113-0·201) | (0·099-0·151) |

**Source:** Author’s data analysis for 40,768 observations – 2,548 municipalities in Brazil over 16 years (from 2004 to 2019).

**Note**: Data are in Rate Ratio (RR) coefficients (95% CI) unless otherwise specified; Time shocks are controls for specific years of economic crisis – 2008, 2013, and 2015; ***, ** and * denote significance at 1%, 5%, and 10% respectively. BFP=Bolsa Familia Program. PBC=Benefício de Prestação Continuada Program.

**Web Table 13 – Empirical results for mortality models with region(state) control, 2004-2019, Brazil.**

|  | **Under 1** | | **From 1 to 4** | | **Under 5** | |
| --- | --- | --- | --- | --- | --- | --- |
|  | Crude | Adjusted | Crude | Adjusted | Crude | Adjusted |
| BFP target coverage |  |  |  |  |  |  |
| Low (0-30%) | 1 | 1 | 1 | 1 | 1 | 1 |
| Intermediate (30-70%) | 1,006 | 1,007 | 1,063 | 1·067* | 1,015 | 1,015 |
|  | (0·974,1·039) | (0·975,1·040) | (0·988,1·143) | (0·992,1·147) | (0·984,1·046) | (0·985,1·046) |
| High (70-100%) | 0·937*** | 0·952*** | 0·959 | 0·980 | 0·940*** | 0·956*** |
|  | (0·907,0·967) | (0·921,0·982) | (0·892,1·031) | (0·912,1·054) | (0·913,0·969) | (0·928,0·985) |
| Consolidated (≥100%) | 0·828*** | 0·868*** | 0·854*** | 0·914** | 0·833*** | 0·875*** |
|  | (0·804-0·854) | (0·842-0·895) | (0·798-0·915) | (0·853-0·979) | (0·810-0·857) | (0·850-0·900) |
| BPC |  |  |  |  |  |  |
| Low (0-33) | 1 | 1 | 1 | 1 | 1 | 1 |
| Intermediate (33-66) | 0·907*** | 0·920*** | 0·895*** | 0·912*** | 0·905*** | 0·918*** |
|  | (0·895-0·920) | (0·907-0·932) | (0·867-0·924) | (0·883-0·942) | (0·893-0·916) | (0·907-0·930) |
| Consolidated (66-100) | 0·791*** | 0·843*** | 0·755*** | 0·822*** | 0·785*** | 0·839*** |
|  | (0·776-0·807) | (0·826-0·860) | (0·721-0·790) | (0·784-0·862) | (0·771-0·799) | (0·824-0·856) |
| Proportion of individuals older than 15 years who are illiterate (%) |  | 1·052*** |  | 1·062** |  | 1·053*** |
|  |  | (1·030-1·075) |  | (1·012-1·114) |  | (1·032-1·074) |
| Poverty rate (%) |  | 1·035*** |  | 1·067*** |  | 1·039*** |
|  |  | (1·019-1·051) |  | (1·029-1·107) |  | (1·024-1·055) |
| Urbanization rate (%) |  | 0·933*** |  | 0·924** |  | 0·932*** |
|  |  | (0·905-0·961) |  | (0·864-0·990) |  | (0·906-0·958) |
| Fertility rate (%) |  | 1·059*** |  | 1·040* |  | 1·057*** |
|  |  | (1·040-1·079) |  | (0·997-1·085) |  | (1·039-1·075) |
| Rate of hospital beds per 1,000 population (%) |  | 1-001 |  | 1-001 |  | 1-001 |
|  |  | (0·983-1·020) |  | (0·960-1·044) |  | (0·984-1·019) |
| Rate of physicians per 1,000 population (%) |  | 0·984* |  | 1-009 |  | 0·987 |
|  |  | (0·966-1·001) |  | (0·970-1·050) |  | (0·971-1·004) |
| Rate of nurses per 1,000 population (%) |  | 0·932*** |  | 0·898*** |  | 0·927*** |
|  |  | (0·920-0·944) |  | (0·872-0·925) |  | (0·916-0·938) |
| Households with proper garbage collection (%) |  | 0·965*** |  | 0·929*** |  | 0·960*** |
|  |  | (0·945-0·986) |  | (0·885-0·976) |  | (0·941-0·979) |
| **Regional binaries** | **Yes** | **Yes** | **Yes** | **Yes** | **Yes** | **Yes** |
| Year binaries | Yes | Yes | Yes | Yes | Yes | Yes |
| AIC | 134,979·9 | 134,562·4 | 640,55·5 | 63,921·6 | 142,235·4 | 141,707·0 |
| BIC | 135,091·9 | 134,743·3 | 641,66·8 | 64,101·4 | 142,347·4 | 141,887·9 |
| Log Likelihood | -67,476·9 | -67,260·2 | -32,014·8 | -31,939·8 | -71,104·7 | -70,832·5 |

**Note**: Data are in Rate Ratio (RR) coefficients (95% CI) unless otherwise specified; Time shocks are controls for specific years of economic crisis – 2008, 2013, and 2015; ***, ** and * denote significance at 1%, 5%, and 10% respectively. BFP=Bolsa Familia Program. PBC=Benefício de Prestação Continuada Program.

**Web Table 14 – Correlations Matrix of the parameter estimates of the main model.**

| e(V) | **BFP_2** | **BFP_3** | **BFP_4** | **BPC_2** | **BPC_3** | **Illitera-cy rate** | **Poverty** | **Urbaniza-tion rate** | **Fertili-ty rate** | **Doctor rate** | **Hospital bed rate** | **Year (2008)** | **Year (2013)** | **Year (2015)** | **_cons** |
| --- | --- | --- | --- | --- | --- | --- | --- | --- | --- | --- | --- | --- | --- | --- | --- |
| childmort |  |  |  |  |  |  |  |  |  |  |  |  |  |  |  |
| BPF_2 (intermediate) | 1.00 |  |  |  |  |  |  |  |  |  |  |  |  |  |  |
| BPF_3 (High) | 0.80 | 1.00 |  |  |  |  |  |  |  |  |  |  |  |  |  |
| BPF_4 (Consolidated) | 0.86 | 0.87 | 1.00 |  |  |  |  |  |  |  |  |  |  |  |  |
| BPC_2 (Intermediate) | 0.00 | -0.04 | -0.11 | 1.00 |  |  |  |  |  |  |  |  |  |  |  |
| BPC_3 (Consolidated) | 0.00 | -0.04 | -0.14 | 0.68 | 1.00 |  |  |  |  |  |  |  |  |  |  |
| Illiteracy rate | 0.02 | 0.03 | 0.05 | -0.01 | 0.02 | 1.00 |  |  |  |  |  |  |  |  |  |
| Poverty rate | 0.01 | 0.03 | 0.07 | 0.07 | 0.09 | -0.20 | 1.00 |  |  |  |  |  |  |  |  |
| Urbanization rate | 0.01 | 0.01 | 0.00 | 0.00 | -0.03 | 0.03 | 0.05 | 1.00 |  |  |  |  |  |  |  |
| Fertility rate | 0.00 | 0.01 | 0.03 | 0.05 | 0.09 | -0.22 | -0.25 | 0.04 | 1.00 |  |  |  |  |  |  |
| Doctor rate | 0.01 | 0.02 | 0.01 | -0.02 | -0.05 | 0.05 | 0.01 | -0.04 | 0.02 | 1.00 |  |  |  |  |  |
| Hospital bed rate | 0.00 | -0.01 | -0.04 | -0.03 | -0.07 | 0.13 | 0.12 | -0.22 | 0.19 | -0.04 | 1.00 |  |  |  |  |
| Year (2008) | 0.00 | -0.04 | -0.09 | 0.08 | 0.12 | -0.04 | 0.05 | 0.01 | -0.08 | 0.02 | 0.04 | 1.00 |  |  |  |
| Year (2013) | 0.00 | 0.00 | -0.04 | -0.02 | -0.03 | -0.02 | 0.07 | 0.01 | 0.00 | 0.05 | -0.01 | 0.08 | 1.00 |  |  |
| Year (2015) | 0.00 | 0.00 | -0.02 | -0.04 | -0.07 | -0.02 | 0.06 | 0.00 | 0.03 | -0.01 | -0.02 | 0.06 | 0.09 | 1.00 |  |
| _cons | -0.64 | -0.64 | -0.70 | -0.16 | -0.15 | -0.23 | -0.22 | -0.21 | -0.20 | -0.14 | -0.19 | 0.00 | -0.02 | -0.01 | 1.00 |

**Source:** Author’s data analysis for 40,768 observations – 2,548 municipalities in Brazil over 16 years (from 2004 to 2019).

**Note**: Data are in correlation coefficients; Time shocks are controls for specific years of economic crisis – 2008, 2013, and 2015. BFP=Bolsa Familia Program. PBC=Benefício de Prestação Continuada Program.

# PART III – FORECASTING ANALYSIS

# Description of the forecasting methodology

The following section provides details of the forecasting process in accordance with standard international modeling reporting guidelines (ISPOR-SMDM). The modeling approach adopted for this study was developed based on two stages.

In the first stage, a synthetic cohort of all Brazilian municipalities for the period 2020-2030 was created as an extension of a longitudinal dataset of 5507 municipalities for 2000-2019 obtained from the sources detailed in Web Table 1. Simulated municipality-specific trends for poverty rates and the other demographic and socioeconomic variables were obtained according to economic crisis scenarios for the years 2020-2030. BFP and BPC coverage were simulated according to social protection policy response scenarios options.

In the second stage, for each year and each municipality, the mortality rate for all the municipalities was estimated as the outcome of the same multivariate fixed effects regressions, using the forecast demographic, socioeconomic and exposure variables (BFP and BPC coverage) as input values.

# Purpose of the forecasting and its applications

The developed model had the overall purpose to simulate the effects of socioeconomic and policy coverage changes on health outcomes in Brazil using ecologic-level data and - when available - retrospective ecologic datasets. Elements of flexibility have been introduced in the code to allow simulation of different sets of variables and different regression models.

# Inputs, outputs, and other parameters

## *7.1.* *Scenarios of poverty and coverage of social welfare programs*

In order to develop forecasting, exponential functions were used to simulate the covariates behavior for the next 11 years (2020-2030). Regarding the poverty rate, an increasing scenario was considered for the first years (economic crisis period). This is described by the equation,

$$x_{t}= x_{2019} + c_{1}x_{2019}\left( 1-\exp\left( -k_{1}t \right) \right), \left( 2 \right)$$

for the remaining years, we consider the exponential decay,

$$x_{t}= x_{2019} - c_{2}x_{2019}\left( 1-\exp\left( -k_{2}t \right) \right), \left( 3 \right)$$

where the parameters $c_{1}$, $k_{1}$, $c_{2}$, $k_{2}$ were settled according to different available sources.

With respect to the intervention variables (BFP and BPC) under the mitigation scenario, they were considered as having the same behavior of the poverty rate (mitigation effect) during the economic crisis^3^. For the post - crisis, the interventions were simulated using the exponential decay in equation (3). The decreasing rate k2 in this case, was settled as half the poverty to simulate the transition period between the crisis and recovery scenarios.

Under the austerity scenarios, the interventions xt, austerity were considered to follow an exponential decay which shows directly the percentage of decrease per year, this allowed to support the policies simulation according to situations that concerns the Brazilian government expenditure.^17^ The equation below describes this dynamic.

$$x_{t,aust} = x_{t,aust} \left( 1-p \right)^{t}$$

where $p$ is the percentage of decreasing for each intervention and t refers to the year.

We simulated three economic crisis scenarios using the increase in the poverty rates, which was calculated using the microdata from the Brazilian National Household surveys from (PNAD) 2004 to 2019, and the special PNAD conducted during the COVID-19 pandemic for the year 2020. Poverty is defined as the percentage of households below the national poverty line, which is also used as an eligibility condition for the Bolsa Familia Conditional Cash Transfer program. The magnitude of the economic crisis is represented by the percentage variation of the poverty rate from 2019 to 2020 without considering the COVID Emergency cash transfer (*Auxílio Emergencial*), hence the COVID10-related economic crisis. Recent reports show that the acute increase of poverty rate from 2020 to 2021 (about 22,7%) is significantly higher compared to the annual poverty increase used as economic crises scenarios in this study.^18^

The economic crisis scenarios considered in this analysis were simulated as follows:

- Shorter Economic Crisis scenario: A milder and shorter economic crisis, with an increase in poverty rates for the first three years (2020 - 2022). This behavior was generated using equation (2), with parameters c1= 0.6, k1 =0.1 .  On the other side, For the post-crisis period (2023 -2030), poverty rates were simulated by using equation (3) with parameters c2 = 0.53 e k2=0.2 .
- Medium Economic Crisis scenario: A medium economic crisis with a larger increase in the poverty rate for the first 5 years (2020-2024). This behavior was generated using equation (2), with parameters c1= 1.2, k1 =0.2 . As in the first scenario, For the post-crisis period (2025 -2030), the poverty rates were simulated by using equation (3) with parameters c2 = 0.53 e k2=0.2.
- Longer Economic Crisis scenario: A longer economic crisis was created using similar parameters as the Medium Economic Crisis scenarios, but with an increase sustained over 7 years (from 2015 to 2021).

In response to the economic crisis, three policy responses were considered in the main analysis:

- Mitigation scenario: a mitigation strategy with a proportional behavior of the BFP and BPC to the poverty scenarios, during the corresponding simulated economic crisis. In this case, these interventions were generated in the same way as poverty rates, considering the same equation and parameters according to each period and scenario.
- Baseline scenario: derived from a validated model - already employed in previous studies^5,6^ - that projected the effects of the current fiscal austerity measures due to the *Emenda Constitucional* 95 (EC95) on the coverage of the three interventions. This scenario was simulated according to the equation (4) considering a percentage of decrease of 5%, as in previous studies.^5,6^
- Severe Austerity scenario: based on the reduction of BFP and BPC proportional to the reduction of government expenditure on social protection (excluding cash transfer programs) observed from 2014-2019 24. This scenario was simulated according to the equation (4) considering a percentage of decrease of 9.8%. This percentage was derived from the reduction of government expenditure on social protection (excluding cash transfer programs) observed from 2014-2019.^24^

The Web Table 14 presents a summary of these scenarios and parameters, while the Web Figure 8 show the behavior of the poverty scenarios.

**Web Table 15– Forecasting scenarios and parameters**

|  | **Economic crisis Scenario** | | |
| --- | --- | --- | --- |
|  | **Shorter Economic Crisis** | **Medium Economic Crisis** | **Longer Economic Crisis** |
| **Parameters** |  |  |  |
| First years | c1= 0.6, k1 =0.1 (from 2000 to 2022) | c1= 1.2, k1 =0.2 (from 2000 to 2024) | c1= 1.2, k1 =0.2 (from 2015 to 2021) |
| Last years | c2 = 0.53 e k2=0.2 (from 2023 to 2030) | c2 = 0.53 e k2=0.2 (from 2025 to 2030) | c2 = 0.53 e k2=0.2. (from 2022 to 2030) |
| **Policy response** |  |  |  |
| Mitigation | generated in the same way as poverty rates | generated in the same way as poverty rates | generated in the same way as poverty rates |
| Baseline | decrease of 5% | decrease of 5% | decrease of 5% |
| Severe Austerity | decrease of 9.8%. | decrease of 9.8%. | decrease of 9.8%. |

**Web Figure 8. Forecasted poverty rate.**


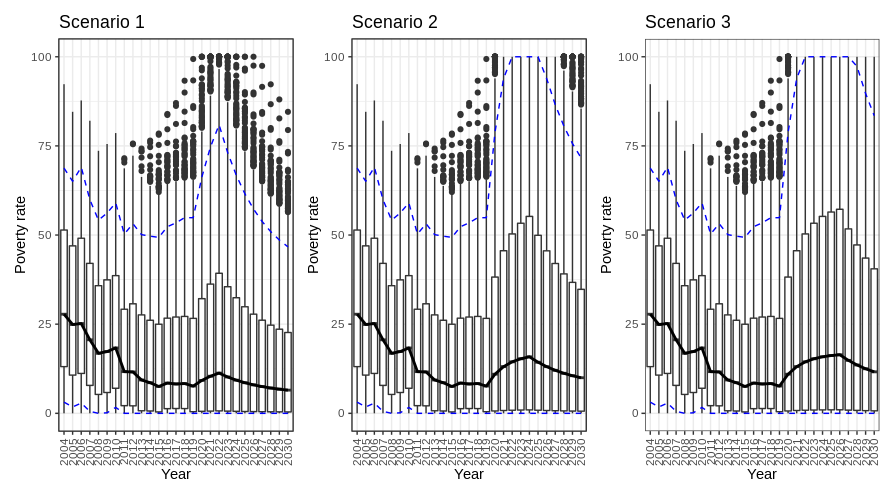


# Prediction methodology

To generate predictions and confidence intervals for each response $Y_{it}$, the Monte Carlo methodology was used. This procedure allows to get more accurate results compared with conventional methods such as the use of the normal distribution. It can be summarized in the following steps

1. Simulate the intervention values for the forecasting period (2020 -2030) using the mitigation and austerity scenarios settled in previous sections. Also simulate the control covariates using equations (2) and (3) and following their trend.
2. Simulate a new $Y_{it}$ from the negative binomial distribution using the estimated parameters from the retrospective study and the forecasted covariates.
3. Get the predictions $E\left( Y_{it} | X \right)$ using the new simulated variable $Y_{it}$, here $X$ represents the set of covariates including the interventions.
4. Get back to step 1.

The algorithm ended when the number of desired Monte Carlo simulations M is reached. The predictions and confidence interval estimated for Y*_it_* will be the mean and the percentiles 2.5% and 97.5% of the M simulations respectively. For each outcome and each scenario, 10,000 simulations were performed, allowing parameter values to vary in each simulation cycle according to their assumed underlying distribution. The number 10,000 was chosen based on the stabilization of the estimates.

## ***8.1.*** ***External validation***

The external validation of the model was undertaken comparing the overall national mortality rate (computed for each municipality) forecasted using microsimulations, with the official Brazilian mortality estimates (overall) during the years 2010-2019, which are the most up-to-date available, and estimating the linear regression and the correlation coefficients (R2) of predicted vs observed values, as shown in Web Figure 9.

**Web Figure 9. Linear regression and correlation coefficient (R2) of predicted vs observed values, and trend of the simulated overall mortality rate vs the official Brazilian mortality rate estimates for the period 2010-2019.**


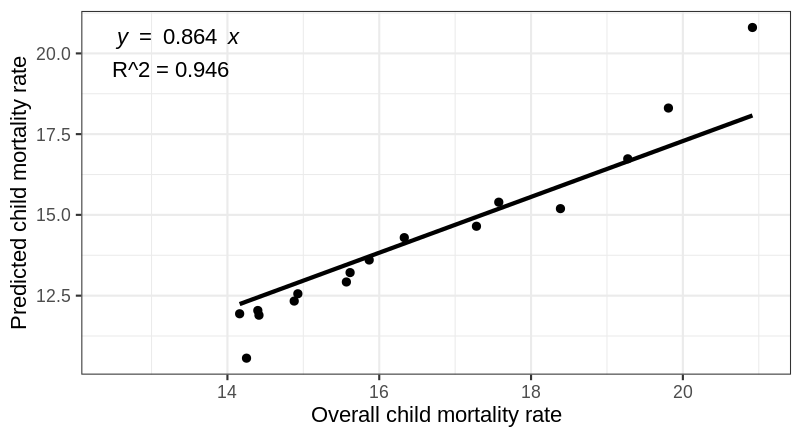


# Sensitivity analysis

To evaluate how a lengthening of the economic crisis could affect mortality rates we additionally modelled the impact of austerity and social protection mitigation considering two additional poverty scenarios (see Web Figures 1, 2 and 3). The Web Table 16 is consistent with the results found for the poverty scenario 2 (see the main manuscript) showing that the averted deaths decrease as the policies conditions improve in terms of increase the coverage of the different social programs

**Web Table 16 – Rate ratio (RR) and cumulative difference in overall deaths over the period 2020-2030 between alternative policy scenarios, according to different the Economic Crisis Scenario.**

|  | **Mitigation vs Baseline (5%)** | | **Mitigation vs Austerity (9.8%)** | |
| --- | --- | --- | --- | --- |
| **Year** | **Rate Ratio (RR)** | **Li -Ls** | **Rate Ratio (RR)** | **Li -Ls** |
| Overall | | | | |
| *Poverty scenario 1* | | | | |
| 2020 | 0·929 | 0·909 - 0·950 | 0·916 | 0·898 - 1 |
| 2025 | 0·840 | 0·800 - 0·883 | 0·756 | 0·706 - 0·840 |
| 2030 | 0·810 | 0·764 - 0·859 | 0·699 | 0·644 - 0·725 |
| Avoidable deaths | 75,332·41 | 62,581·74 - 88,194·31 | 149,868·92 | 128,130·73 - 171,928·78 |
| *Poverty scenario 2* | | | | |
| 2020 | 0·906 | 0·881 - 0·933 | 0·893 | 0·869 - 0·911 |
| 2025 | 0·803 | 0·761 - 0·847 | 0·721 | 0·669 - 0·750 |
| 2030 | 0·761 | 0·714 - 0·811 | 0·653 | 0·596 - 0·696 |
| Avoidable deaths | 87,808 | 73,096 - 102,661 | 148,736 | 127,148 - 170,706 |
| *Poverty scenario 3* | | | | |
| 2020 | 0·906 | 0·881 - 0·933 | 0·893 | 0·869 - 0·911 |
| 2025 | 0·798 | 0·756 - 0·843 | 0·716 | 0·665 - 0·744 |
| 2030 | 0·752 | 0·705 - 0·801 | 0·644 | 0·587 - 0·694 |
| Avoidable deaths | 88,839·93 | 74,003·56 - 103,904·01 | 149,868·92 | 128,130·73 - 171,928·78 |

**Source:** Author's data analysis for 5,507 municipalities in Brazil.

**Note:** Data are in Incidente-Rate Ratio (IRR) coefficients (95% CI) unless otherwise specified. “Li” is inferior limit (or lower limit) and “Ls” is the superior limit (or upper limit) of confidence interval.

# Main limitations

The main limitation of the study is the uncertainty around the future macroeconomic scenarios in Brazil, due to the current extremely unstable political and economic situation, which creates uncertainty around the forecasting of poverty rates, income, and the other independent variables. For that reason, several scenarios have been simulated in sensitivity analyses which produced comparative findings. Another limitation is that the modelling of austerity measures is focused on BFP and BPC as there is strong evidence that these policies confer protective effects for overall and childhood morbidity and mortality from previous studies.^1^

Our estimates of the impact of austerity measures on under 5 age groups are probably conservative as they do not reflect constraints in other areas of public spending e.g., education, housing and other welfare programs which have known impacts on poverty and health. Moreover, austerity measures recently enshrined in the constitution of Brazil means that public spending will only increase in line with inflation, which will not account for the demographic growth of the population, its ageing processes, and growing costs associated with new healthcare treatments and technologies.^19–21^ Another limitation of the study is that we do not model the impact of the increased coverage of BFP on poverty rate dynamics, assuming that poverty rates influence BFP coverage and not the contrary.

This is mainly due to the assumption that WB simulations of poverty increase during economic crisis already account for BFP effects, and because reliable parameters were not available at the moment of writing.

# REFERENCES

1 Rasella D, Aquino R, Santos CAT, Paes-Sousa R, Barreto ML. Effect of a conditional cash transfer programme on childhood mortality: A nationwide analysis of Brazilian municipalities. *The Lancet* 2013; **382**. DOI:10.1016/S0140-6736(13)60715-1.

2 Lucas ADP, de Oliveira Ferreira M, Lucas TDP, Salari P. The intergenerational relationship between conditional cash transfers and newborn health. *BMC Public Health* 2022; **22**. DOI:10.1186/s12889-022-12565-7.

3 Rasella D, Basu S, Hone T, Paes-Sousa R, Ocké-Reis CO, Millett C. Child morbidity and mortality associated with alternative policy responses to the economic crisis in Brazil: A nationwide microsimulation study. *PLoS Med* 2018; **15**. DOI:10.1371/journal.pmed.1002570.

4 Rasella D, Alves FJO, Rebouças P, *et al.* Long-term impact of a conditional cash transfer programme on maternal mortality: a nationwide analysis of Brazilian longitudinal data. *BMC Med* 2021; **19**. DOI:10.1186/s12916-021-01994-7.

5 Ramos D, da Silva NB, Ichihara MY, *et al.* Conditional cash transfer program and child mortality: A cross-sectional analysis nested within the 100 Million Brazilian Cohort. *PLoS Med* 2021; **18**. DOI:10.1371/journal.pmed.1003509.

6 Aguila E, López-Ortega M, Robledo LMG. Non-contributory pension programs and frailty of older adults: Evidence from Mexico. *PLoS One* 2018; **13**. DOI:10.1371/journal.pone.0206792.

7 Riumallo-Herl C, Aguila E. The effect of old-age pensions on health care utilization patterns and insurance uptake in Mexico. *BMJ Glob Health* 2019; **4**. DOI:10.1136/bmjgh-2019-001771.

8 Hessel P, López LC, Ordóñez-Monak I, González-Uribe C. The relation between social pensions and health among poor older individuals in Colombia: A qualitative study. *Ageing Soc* 2020. DOI:10.1017/S0144686X20001361.

9 Bertranou FM, Van Ginneken W, Solorio C. The impact of tax-financed pensions on poverty reduction in Latin America: Evidence from Argentina, Brazil, Chile, Costa Rica and Uruguay. *Int Soc Secur Rev* 2004; **57**. DOI:10.1111/j.1468-246X.2004.00200.x.

10 Huang W, Zhang C. The Power of Social Pensions: Evidence from China’s New Rural Pension Scheme. *Am Econ J Appl Econ* 2021; **13**: 179–205.

11 Duflo E. Child outcomes in Africa: Child health and household resources in South Africa: Evidence from the old age pension program. *American Economic Review* 2000; **90**. DOI:10.1257/aer.90.2.393.

12 Duflo E. Grandmothers and granddaughters: Old-age pensions and intrahousehold allocation in South Africa. *World Bank Economic Review* 2003; **17**. DOI:10.1093/wber/lhg013.

13 Ponczek V. Income and bargaining effects on education and health in Brazil. *J Dev Econ* 2011; **94**. DOI:10.1016/j.jdeveco.2010.01.011.

14 Hone T, Mirelman AJ, Rasella D, *et al.* Effect of economic recession and impact of health and social protection expenditures on adult mortality: a longitudinal analysis of 5565 Brazilian municipalities. *Lancet Glob Health* 2019; **7**. DOI:10.1016/S2214-109X(19)30409-7.

15 Lawlor DA, Tilling K, Davey Smith G. Triangulation in aetiological epidemiology. *Int J Epidemiol* 2017; : dyw314.

16 Khandker S, B. Koolwal G, Samad H. Handbook on Impact Evaluation. The World Bank, 2009 DOI:10.1596/978-0-8213-8028-4.

17 Mariani, C. B. Gomes, E. C. Cenci, D. R. Queiroz RF de. Financiamento da Assistência Social no Brasil Nota Técnica de Monitoramento (2019). 2019.

18 Salata ARicardo, Ribeiro MGomes. Boletim Desigualdade nas Metrópoles (n. 09). Observatório das Metrópoles. Porto Alegre/RS, 2022.

19 Paiva AB, Mesquita ACS, Jaccoud L, Passos L. [The new tax regime and its implications for social assistance policy in Brazil.] [Portuguese]. *Instituto de Pesquisa Econômica Aplicada* 2016; **27**.

20 Vieira, F. S. Benevides RPDS. [The new tax regime and its implications for social assistance policy in Brazil.] [Portuguese]. Technical Note No.27. 2016.

21 Rossi P, Dweck E. Impacts of the New Fiscal Regime on health and education Impactos. *Cad Saude Publica* 2016; **32**.
